# *N* ^6^‐methyladenosine‐modified *circSTX6* promotes hepatocellular carcinoma progression by regulating the HNRNPD/*ATF3* axis and encoding a 144 amino acid polypeptide

**DOI:** 10.1002/ctm2.1451

**Published:** 2023-10-25

**Authors:** Jiahua Lu, Junnan Ru, Yunhao Chen, Zhenan Ling, Hanqing Liu, Bo Ding, Yifan Jiang, Jun Ma, Deguo Zhang, Jiangzhen Ge, Yu Li, Fei Sun, Diyu Chen, Shusen Zheng, Jian Wu

**Affiliations:** ^1^ Division of Hepatobiliary and Pancreatic Surgery Department of Surgery The First Affiliated Hospital Zhejiang University School of Medicine Hangzhou Peoples Republic of China; ^2^ NHC Key Laboratory of Combined Multi‐organ Transplantation Hangzhou Peoples Republic of China; ^3^ Key Laboratory of the diagnosis and treatment of organ Transplantation Research Unit of Collaborative Diagnosis and Treatment For Hepatobiliary and Pancreatic Cancer Chinese Academy of Medical Sciences (2019RU019) Hangzhou Peoples Republic of China; ^4^ Key Laboratory of Organ Transplantation Research Center for Diagnosis and Treatment of Hepatobiliary Diseases Hangzhou Zhejiang Province Peoples Republic of China; ^5^ Department of Thoracic Surgery The First Affiliated Hospital Zhejiang University School of Medicine Hangzhou Peoples Republic of China

**Keywords:** circRNA, hepatocellular carcinoma, *N* ^6^‐methyladenosine modification, mRNA decay, protein encoding, RNA‐binding protein

## Abstract

**Background:**

Circular RNAs (circRNAs) play a significant role in the initiation and progression of various cancers, including hepatocellular carcinoma (HCC). *Circular syntaxin 6* (*circSTX6*, also known as *hsa_circ_0007905*) has been identified as a microRNA (miRNA) sponge in pancreatic adenocarcinoma. However, its full range of functions in terms of protein scaffold and translation remain largely unexplored in the context of HCC.

**Methods:**

The expression of *circSTX6* and its encoded protein was examined in HCC tumour tissues. *N* ^6^‐methyladenosine (m^6^A) on *circSTX6* was verified and quantified by methylated RNA immunoprecipitation (Me‐RIP), RIP and dual luciferase reporter assays. The biological functions of *circSTX6* and its encoded protein in HCC were clarified by in vitro and in vivo experiments. Mechanistically, the interaction between *circSTX6* and heterogeneous nuclear ribonucleoprotein D (HNRNPD) was investigated by RNA pull‐down, RIP and fluorescence in situ hybridization (FISH)/IF. The regulatory effects of *circSTX6* and HNRNPD on *activating transcription factor 3* (*ATF3*) mRNA were determined by mRNA stability and RIP assays. Furthermore, the presence of *circSTX6*‐encoded protein was verified by mass spectrometry.

**Results:**

*CircSTX6* and its encoded 144 amino acid polypeptide, circSTX6‐144aa, were highly expressed in HCC tumour tissues and served as independent risk factors for overall survival in HCC patients. The expression of *circSTX6* was regulated by METTL14 in an m^6^A‐dependent manner. Functionally, *circSTX6* accelerated HCC proliferation and tumourigenicity and reinforced tumour metastasis in vitro and in vivo. Mechanistically, *circSTX6* acted as a sponge for HNRNPD protein, facilitating its binding to *ATF3* mRNA, consequently promoting *ATF3* mRNA decay. Meanwhile, circSTX6‐144aa promoted HCC proliferation, migration and invasion independent of *circSTX6* itself.

**Conclusion:**

Collectively, our study reveals that m^6^A‐modified *circSTX6* drives malignancy in HCC through the HNRNPD/*ATF3* axis, while its encoded circSTX6‐144aa contributes to HCC progression independent of *circSTX6*. *CirSTX6* and its encoded protein hold promise as potential biomarkers and therapeutic targets in HCC.

## Graphical abstract

- METTL14‐mediated *N* ^6^‐methyladenosine (m^6^A) modification on *circSTX6* inhibits its expression.
- *CircSTX6* promotes proliferation and metastasis of hepatocellular carcinoma (HCC) by binding to HNRNPD to facilitate HNRNPD‐mediated *ATF3* mRNA degradation.
- Driven by IRES1, *circSTX6* encoded a 144 amino acid novel polypeptide, circSTX6‐144aa, which promotes HCC progression independent of *circSTX6* itself.

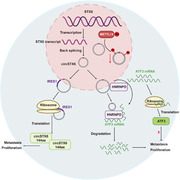

## 1 BACKGROUND

Hepatocellular carcinoma (HCC) is the third leading cause of cancer‐related deaths globally, with an estimated 830, 000 fatalities annually.^1^ Despite significant advancements in diagnostic methods, surgical techniques and the emergence of targeted therapies and immunotherapies in recent years, the long‐term overall prognosis for HCC patients remains unsatisfactory.^2^, ^3^ Challenges persist due to the high rates of metastasis and recurrence, largely attributed to an incomplete understanding of tumour pathogenesis.^4^ Thus, there is an imperative need to identify novel therapeutic targets and gain a deeper insight into the molecular mechanisms governing HCC for enhanced treatment efficacy.

Circular RNAs (circRNAs), a unique subclass of endogenous noncoding RNAs (ncRNAs) characterized by their covalently closed loop structures, have attracted considerable attention.^5^ CircRNAs exhibit evolutionary conservation, tissue‐specific expression and enhanced stability owing to their circularized structure.^5^, ^6^ A mounting body of evidence suggested that circRNAs actively participate in the tumourigenesis and metastasis of cancers, and its role as miRNA sponge has been widely reported in HCC since its discovery.^7^, ^8^ For instance, *circASAP1* promotes HCC pulmonary metastasis by sponging *miR‐532‐5p* and *miR‐326* that mediate MAPK1 and CSF‐1 signalling pathways.^9^ Notably, increasing studies are focusing on circRNAs beyond their function as miRNA sponges. Our previous study demonstrated that circCPSF6 competitively binds to an RNA‐binding protein (RBP) to activate Yes‐associated protein (YAP) signalling and drive HCC malignancies.^10^ Moreover, studies related to the “unconventional” roles of circRNAs regarding protein encoding are emerging. Recently, a novel secretory polypeptide encoded by *circZKSCAN1* was reported to suppress HCC progression through the degradation of mammalian targets of rapamycin (mTOR).^11^ Thus, additional efforts are needed to unveil the roles of circRNAs in HCC beyond the miRNA sponge.

*N* ^6^‐methyladenosine (m^6^A) as one of the most prevalent epigenetic modifications in eukaryotic RNAs, is catalyzed by methyltransferases such as methyltransferase‐like protein 3 (METTL3), METTL14, and Wilm's tumour 1‐associated protein (WTAP), recognized by m^6^A binding proteins such as YTH domain‐containing family proteins (YTHDF) and insulin‐like growth factor 2 mRNA‐binding proteins (IGF2BP) and removed by demethylases including fat mass and obesity‐associated protein (FTO) and AlkB homolog 5 (ALKBH5).^12^ m^6^a modification regulates RNA metabolism in diverse ways, including export, splicing, degradation, stability and translation.^13^ Intriguingly, accumulating studies have established a relationship between circRNAs and m^6^A.^14^ For example, m^6^A can regulate circRNA degradation through YTHDF2‐mediated endoribonuclease cleavage.^15^ Similarly, ALKBH5‐mediated m^6^A modification on *circCCDC134* increases its stability, promoting gastric cancer tumour growth and metastasis.^16^ Moreover, *circMAP3K4* prevents HCC apoptosis by encoding circMAP3K4‐455aa protein, whose translation is driven by IGF2BP1 in an m^6^A‐dependent manner.^17^ These findings underscore the essential role of m^6^A in circRNA regulation across multiple cancers, including HCC.^10^, ^18^, ^19^ Nonetheless, the functions of m^6^A‐regulated circRNAs in HCC and their underlying mechanisms demand further investigation, and it would be important to understand the metabolism and functions of circRNAs by revealing whether and how they are regulated by m^6^A modification as well as to investigate novel therapeutics.

In this study, we identified *circSTX6* by taking the intersection of circRNAs associated with m^6^A and HCC. The expression of *circSTX6* was regulated by METTL14‐mediated m^6^A modification, and *circSTX6* was prevalently upregulated in tumour tissues, correlating with adverse outcomes in HCC patients. Functionally, *circSTX6* promoted cell proliferation, migration and invasion in vitro and accelerated tumour growth and metastasis in vivo. Mechanistically, *circSTX6* bound to heterogeneous nuclear ribonucleoprotein D (HNRNPD) to enhance mRNA decay of *activating transcription factor 3* (*ATF3*), thus inhibiting its expression and facilitating HCC development. Furthermore, *circSTX6* encoded a novel 144 amino acid (aa) polypeptide that accurately predicted poor prognosis in HCC. CircSTX6‐144aa promoted tumour progression independent of *circSTX6* itself. In sum, our study expands the knowledge of m^6^A‐guided regulation of *circSTX6*, reveals its novel mechanisms in HCC tumourigenesis and demonstrates its diagnostic and therapeutic potential.

## 2 MATERIALS AND METHODS

### 2.1 Clinical specimens and grouping

Surgical specimens of 192 HCC patients who underwent radical resection (no prior chemotherapy or radiotherapy) were obtained from The First Affiliated Hospital, Zhejiang University School of Medicine, between 2015 and 2019. This study was conducted with the approval of the Research Ethics Committee of the First Affiliated Hospital of Zhejiang University School of Medicine, and all procedures were in accordance with the guidelines of the Declaration of Helsinki (1975). HCC cohort‐1 consisted of 72 HCC patients. The corresponding frozen tumour tissues were used for RNA extraction and quantitative real‐time PCR (qPCR). The RNA expression of *circSTX6*, *METTL14*, *ATF3* and *circSTX6‐144aa* were determined and correlation analysis was performed. HCC cohort‐2 consisted of 40 HCC patients. Tumour tissues were used for in situ hybridization (ISH) and immunohistochemistry (IHC) staining to explore the correlation between *circSTX6*, METTL14 and ATF3, respectively, and for Western blotting to determine the expression of circSTX6‐144aa in HCC. HCC cohort‐3 consisted of 80 HCC patients, with complete clinicopathological information and follow‐up data. Tumour tissues were also used for constructing HCC tissue microarray, ISH and IHC. The clinical relevance of *circSTX6* and circSTX6‐144aa was analysed separately based on HCC cohort‐3. Patient anthropometrics and demographics for HCC cohort‐1, HCC cohort‐2 and HCC cohort‐3 are provided in Supplementary Tables S1, S2 and S3, respectively.

### 2.2 Cell culture and transfection

The immortalized hepatocyte cell lines (MIHA and HL‐7702), the human HCC cell lines (SK‐Hep‐1, MHCC97H, SNU398, SNU449, HCCLM3, HepG2, Hep3B, Huh7 and PLC/PRF/5) and the human embryonic kidney (HEK)−293 T cells were purchased from the Shanghai Institute of Biochemistry and Cell Biology affiliated to Chinese Academy of Sciences. Cells were cultured in Dulbecco's modified Eagle's medium [Biological Industries (BI)] supplied with 10% foetal bovine serum (BI), 100 μg/mL streptomycin (Sigma‐Aldrich) and 100 U/mL penicillin (Sigma‐Aldrich) at 37°C with a humidified incubator (ThermoFisher) of 5% CO_2_. For all experiments, cells (MIHA, HL‐7702, SK‐Hep‐1, MHCC97H, SNU398, SNU449, HCCLM3, HepG2, Hep3B, Huh7, PLC/PRF/5 and HEK‐293 T) were cultured within 20 passages.

Small interfering RNAs (siRNAs) targeting the junction motifs of *circSTX6* as well as targeting METTL3, METTL14, WTAP, ALKBH5, FTO, HNRNPD and ATF3 were synthesized by Shangya Biotechnology (Hangzhou, China) and Tsingke Biological Technology (Beijing, China). Cells were transfected using the jetPRIME kit (Polyplus, France) according to protocols and collected after 48 h for qPCR and 72 h for Western blot and other assays. Detailed sequences are listed in Supplementary Table S4.

Lentivirus plasmids carrying short hairpin RNA sequences specifically targeting *circSTX6* or negative control RNAs were constructed by RepoBio (Hangzhou, China) as well as lentiviruses expressing *circSTX6* and empty vectors. These plasmids were packaged with pMD2G and psPAX2 and transfected into 293T cells. Lentivirus supernatants were harvested 48 h after transfection and were used for the transfection of target cells. The transfected cells were subject to the filtration of 3 μg/mL puromycin for 1 week and checked for efficiency by qPCR. Similarly, *circSTX6* pcDNA3.1, *circSTX6* with ATG mutation (*circSTX6*‐MUT) pcDNA3.1 and linear circSTX6‐144aa pcDNA3.1 plasmids were constructed. Detailed sequences are listed in the Supplementary Materials and Methods section.

### 2.3 Statistical analysis

GraphPad Prism V8.0.1 software and R V.4.2.2 software were used for statistical analyses, and the results are presented as the means ± standard deviation (SD). The χ2 test was used to assess the qualitative data, and the paired or unpaired two‐tailed Student's *t*‐tests were used for the comparison of quantitative data. The log‐rank tests were used for Kaplan–Meier survival analysis and the Cox regression analysis was used for the identification of independent prognostic factors. The Pearson correlation analysis was conducted between two genes with linear regression. In this study, *p* < 0.05 was defined as statistically significant (*p < 0.05; **p < 0.01; ***p < 0.001). The in vitro assays including colony formation, apoptosis, 5‐Ethynyl‐20‐deoxyuridine (EdU), wound‐healing and transwell assays were conducted in three replicates. The CCK‐8 assay was carried out in five replicates for each time point. Another detailed methodology was described in the Supplementary Materials and Methods section.

## 3 RESULTS

### 3.1 Identification and characterization of *circSTX6* in HCC

**FIGURE 1:**
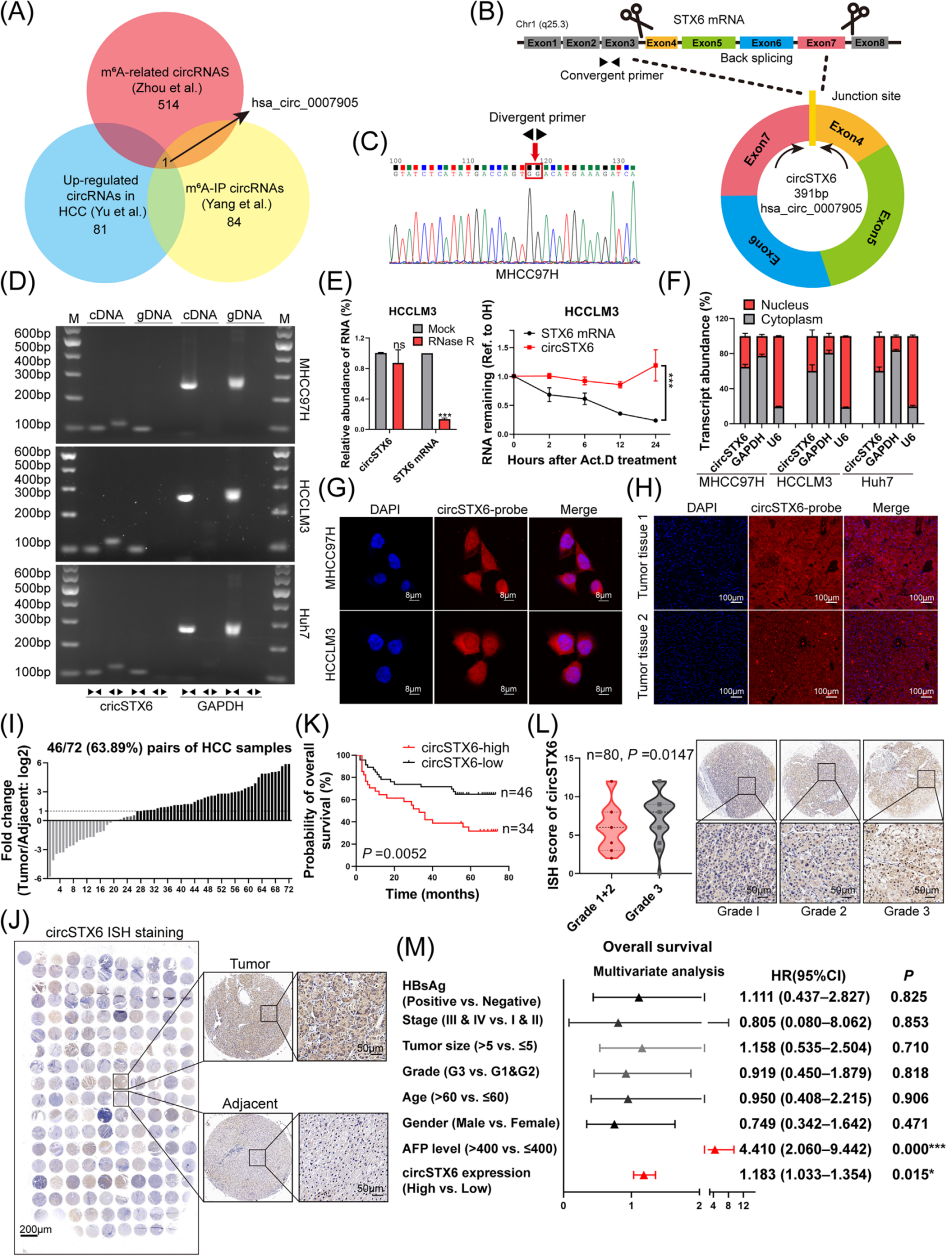
Identification, validation and clinical relevance of *circSTX6* in HCC. (A) Venn diagram showing the overlap of m^6^A‐IP circRNAs (85 transcripts), m^6^A‐related circRNAs (515 transcripts) and upregulated circRNAs (82 circRNAs, fold change ≥2) in HCC. *Hsa_circ_0007905* (*circSTX6*) was the only gene intersected by three datasets. (B) Schematic illustration of the constitution of circSTX6. (C) The back‐splicing junction site was verified by Sanger sequencing in HCC cells. (D) Agarose gel electrophoresis using the PCR products of *circSTX6* and GAPDH amplified by divergent and convergent primers, respectively. (E) The abundance of *circSTX6* and *STX6* mRNA was detected by qPCR after RNase R (3U/ug) or actinomycin D (10 μg/mL) treatment, respectively. (F) Subcellular distribution of *circSTX6* was determined by qPCR with nuclear/cytoplasmic fractionation. *GAPDH* and *U6* were chosen for cytoplasmic and nuclear internal references, respectively. (G) FISH assay shows predominantly cytoplasmic localization of *circSTX6* (Cy3 labelled, RED). Scale bar, 8 μm. (H) Representative FISH images of *circSTX6* (Cy3 labelled, RED) in HCC tumour tissues. Scale bar, 100 μm. (I) The expression of *circSTX6* in 72 pairs of HCC (HCC cohort‐1) tumour tissues and adjacent normal tissues was determined by qPCR and is shown in histograms. (J) The overall ISH staining and representative images of *circSTX6* in HCC cohort‐3 are shown. Scale bar in the overall image, 200 μm; scale bar in representative images, 50 μm. (K) Kaplan–Meier prognostic analyses show the OS (*n* = 80) of HCC patients based on *circSTX6* expression. (L) The expression of *circSTX6* was detected in different pathological grades by ISH. Scale bar, 50 μm. (M) Forest plot shows the hazard ratio (HR) and 95% confidence intervals (CI) of risk factors in HCC cohort‐3 based on Cox multivariate analysis. *CircSTX6* high expression and AFP levels are independent risk factors. *p < 0.05; **p < 0.01; ***p < 0.001; ns, not significant.

To identify m^6^A‐regulated circRNAs in HCC, we analysed circRNA‐seq data from three published studies, including datasets of m^6^A‐IP‐identified circRNAs,^20^ upregulated circRNAs in HCC tumour tissues^8^ and genome‐wide mapping of m^6^A‐regulated circRNAs.^21^ As demonstrated in the Venn plot, only one particular m^6^A‐related circRNA was obtained (Figure 1A). *Hsa_circ_0007905* is derived from exons 4, 5, 6 and 7 of the gene *STX6* on human chromosome 1 (q25, 3) (Figure 1B). Divergent and convergent primers were designed, respectively, based on the junction sites and parental genes. The back‐splicing sites between exon 4 and exon 7 were validated by Sanger sequencing in HCC cells using divergent primers (Figures 1C and Supplementary Figure S1A). The expression of *circSTX6* was determined in two hepatocytes and nine HCC cell lines. MHCC97H, HCCLM3 and Huh7 cells were chosen for subsequent studies (Supplementary Figure S1B). Additionally, according to agarose gel electrophoresis results, *circSTX6* could only be amplified by divergent primers from cDNA instead of gDNA (Figure 1D).

The characteristics of *circSTX6* were verified by a series of assays. HCC cells were treated with ribonuclease R (RNase R) and actinomycin D to determine the stability of *circSTX6*. Results showed that *cricSTX6* was more resistant to RNase R and more stable under actinomycin D treatment compared to its parental transcripts (Figure 1E and Supplementary Figure S1C,D). RNA fractionation (nuclear/cytosolic) assay followed by qPCR demonstrated the predominant cytoplasm distribution of *circSTX6* (Figure 1F), and this result was further confirmed by fluorescence in situ hybridization (FISH) assays performed on HCC cells (Figure 1G) and tumour tissues (Figure 1H and Supplementary Figure S1E). These results confirm that *circSTX6* is a genuine circRNA characterized by the expected biological features.

To further evaluate the aberrant expression of *circSTX6* in HCC, qPCR was performed on HCC cohort‐1 with 72 pairs of tumour and adjacent tissues. The results revealed higher expression of *circSTX6* in tumour tissues compared to matched adjacent tissues (Figure 1I and Supplementary Figure S1F). Moreover, the ISH staining assay demonstrated that *circSTX6* was more abundant in tumour tissues of HCC cohort‐3 (Figure 1J and Supplementary Figure S1G), and Kaplan–Meier survival analysis revealed that upregulation of *circSTX6* in HCC patients was associated with worse overall survival (OS; *p* = 0.0052; Figure 1K) and recurrence‐free survival (RFS; *p* = 0.0094) (Supplementary Figure S1H). Notably, high *circSTX6* expression might be associated with advanced tumour stage and higher pathological grade (Figure 1L and Supplementary Figure S1I). Consistently, receiver operating characteristic (ROC) curves showed that the area under curve (AUC) value of *circSTX6* is 0.8565 (*p* < 0.0001), suggesting *circSTX6* could distinguish between HCC tumour and normal tissues (Supplementary Figure S1J). Besides, the clinical pathological characteristics of HCC cohort‐3 were collected and analysed in detail. It was revealed that *cricSTX6* was also significantly correlated with tumour differentiation (*p* = 0.0061), stage (*p* = 0.0331) and tumour size (*p* = 0.0012) (Supplementary Table S3). Additionally, overexpression of *circSTX6* (hazard ratio [HR] = 1.183, *p* = 0.015) as well as AFP (HR = 4.410, *p* < 0.001) could be considered as independent risk factors for HCC overall survival (Figure 1M). Moreover, Kaplan–Meier survival analysis based on the combination of *circSTX6* expression and AFP levels further revealed that HCC individuals with high *circSTX6* expression and high AFP levels had an even worse OS than any other groups (*p* < 0.0001) (Supplementary Figure S1K), especially compared with those of low *circSTX6* expression and low AFP levels. Taken together, the upregulation of *circSTX6* in HCC indicates a poor prognosis and it may serve as a potential biomarker.

### 3.2 *CircSTX6* is regulated by METTL14‐mediated m^6^A modification

**FIGURE 2:**
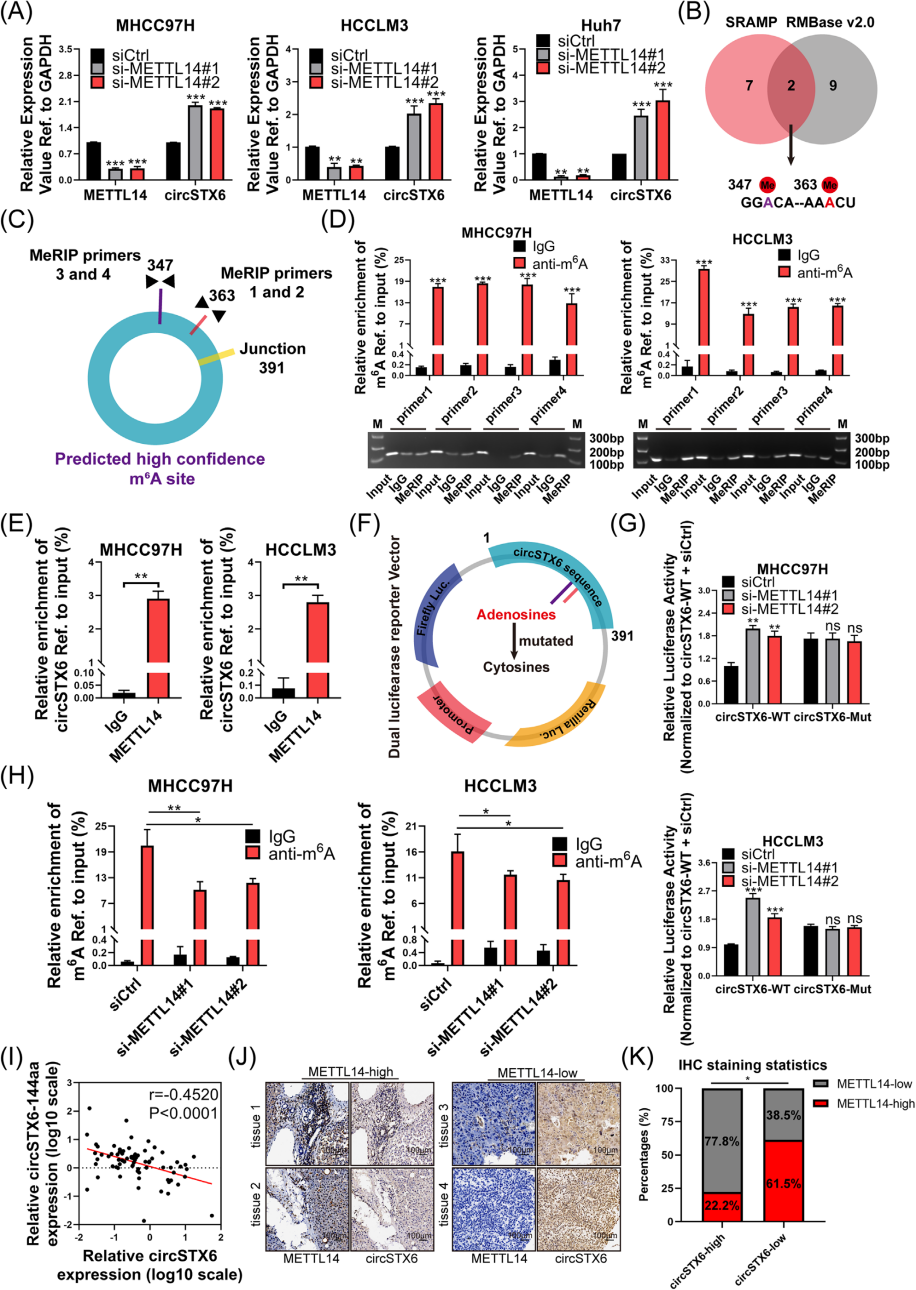
*CircSTX6* is regulated by METTL14‐mediated m^6^A modification. (A) Expression of *circSTX6* upon METTL14 knockdown was detected by qPCR. (B) Potential m^6^A sites of *circSTX6* were predicted by SRAMP and RMBase v 2.0 software. Two sites were obtained from the intersection, one at 347 nt with high confidence (purple) and the other at 363 nt with moderate confidence (red). (C) MeRIP primers were designed to target each site and span the junction. (D) MeRIP assays were conducted in HCC cells and measured by qPCR. Relative enrichment by m^6^A‐antibody was normalized based on input. Results of agarose gel electrophoresis using PCR products are shown. (E) RIP assays followed by qPCR verified the enrichment of *circSTX6* by METTL14 in HCC cells. (F) Schematic illustration of the construction of dual luciferase reporter assays. Sequences of *circSTX6* were cloned into the vector, with (mutant group) or without (wild‐type group) mutating the adenosines into cytosines. (G) Wild‐type or mutant luciferase plasmids and siMETTL14 RNAs were co‐transfected into HCC cells, followed by measurement of the luciferase activities. (H) MeRIP assay results detected by qPCR in METTL14‐knockdown or control HCC cells. (I) Correlation analyses between *circSTX6* and *METTL14* based on qPCR results in HCC cohort‐1 (*n* = 72). (J) ISH staining of *circSTX6* and IHC staining of METTL14 were conducted in HCC cohort‐2 (*n* = 40). Representative images of high‐ and low‐ expression of *circSTX6* and METTL14 are shown, respectively. Scale bar, 100 μm. (K) Statistical analyses based on the IHC/ISH score show the percentages of HCC specimens with high‐ or low *circSTX6*/METTL14 expression. *p < 0.05; **p < 0.01; ***p < 0.001; ns, not significant.

Since *circSTX6* was identified by taking the intersection of two m^6^A‐related datasets, we explored whether *circSTX6* is regulated by m^6^A ‘writers’ or ‘erasers’. Interestingly, the knockdown of METTL14 led to increased *circSTX6* expression in HCC cells (Figure 2A). Whereas inhibition of METTL3, WTAP, ALKBH5 and FTO scarcely influenced the expression of *circSTX6* (Supplementary Figure S2A–D). Thus, we speculated that METTL14 might be the major m^6^A regulator of *circSTX6* expression. Then we used SRAMP^22^ and RMBase v 2.0^23^ to predict potential m^6^A sites on *circSTX6*. Two potential m^6^A sites were obtained by overlapping the results (Figure 2B). Notably, one site was endowed with high confidence (purple) by SRAMP, and the other with moderate confidence (red). The corresponding primers (two pairs of primers for each site) were designed for subsequent MeRIP assays (Figure 2C). Consistently, m^6^A‐modified *circSTX6* RNA fragments were significantly enriched by anti‐m^6^A antibody in MHCC97H, HCCLM3 and Huh7 cells (Figure 2D and Supplementary Figure S2E). Next, we performed RIP assays with anti‐METTL14 antibodies, and the results showed a direct interaction between METTL14 and *circSTX6* RNA (Figure 2E). Furthermore, dual‐luciferase reporter plasmids were constructed by mutating the adenosines into cytosines on *circSTX6* sequences (Figure 2F). The results showed relative luciferase intensity increased upon METTL14 silencing in the wild‐type groups (Figure 2G). Intriguingly, the overall luciferase intensity increased in mutant groups compared with wild‐type groups but was not changed upon METTL14 silencing (Figure 2G), suggesting that METTL14‐mediated regulation of *circSTX6* was m^6^A dependent. Furthermore, MeRIP assays indicated that m^6^A enrichment of *circSTX6* was reduced upon METTL14 knockdown (Figure 2H). Overall, these results demonstrate that the expression of *circSTX6* is at least partially regulated by m^6^A in a METTL14‐dependent manner.

To clarify the clinical relevance between m^6^A and *circSTX6*, MeRIP was conducted on three pairs of HCC tumours and adjacent normal tissues. Notably, one in three tumour tissues demonstrated decreased m^6^A levels of *circSTX6* compared with normal tissues (Supplementary Figure S2F). Additionally, the expression of METTL14 in HCC cohort‐1 was determined by qPCR. The qPCR results revealed that METTL14 was negatively associated with *circSTX6* (*r* = −0.452, *p* < 0.001; Figure 2I). In addition, the ISH staining of *circSTX6* and IHC staining of METTL14 were performed based on HCC cohort‐2 (Figure 2J). ISH and IHC score analyses demonstrated that around 61.5% of specimens that highly expressed METTL14 exhibited weak staining of *circSTX6*, while about 77.8% of specimens with low expression of METTL14 exhibited strong *circSTX6* staining (Figure 2K). Taken together, *circSTX6* is closely associated with m^6^A and is negatively correlated with METTL14 in HCC tumour tissues.

### 3.3 *CircSTX6* accelerates the proliferation of HCC in vitro and in vivo

**FIGURE 3:**
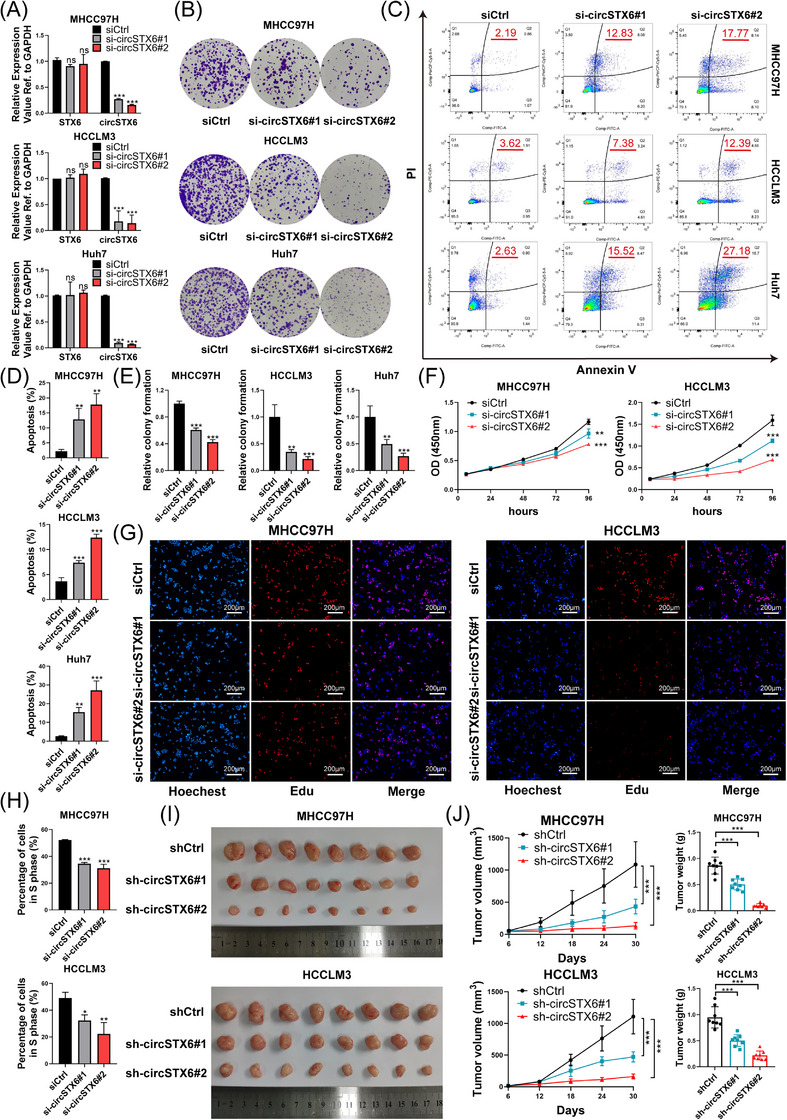
Knockdown of *circSTX6* inhibits HCC proliferation in vitro and in vivo. (A) qPCR analysis detecting the knockdown efficiency of *circSTX6* in MHCC97H, HCCLM3 and Huh7 cells. (B) Colony formation assay was performed to detect the proliferation of HCC cells upon *circSTX6* silencing. (C) Flow cytometry was conducted to evaluate the apoptosis of HCC cells upon *circSTX6* knockdown. HCC cells were stained with Annexin V and propidium iodide. (D and E) The percentages of apoptotic cells and relative colony numbers are quantified in diagrams, respectively. (F) Growth curves in the CCK‐8 assay show the proliferation of HCC cells transfected with *circSTX6* siRNAs. (G and H) Representative images of EdU assays with corresponding quantified bar charts. Scale bar, 200 μm. (I) Representative images of subcutaneously implanted tumors established with MHCC97H and HCCLM3 cells in nude mice (*n* = 8). (J) Statistical analysis of the growth curves and tumour weight of subcutaneous tumours in different groups. *p < 0.05; **p < 0.01; ***p < 0.001; ns, not significant. *p < 0.05; **p < 0.01; ***p < 0.001; ns, not significant.

To investigate the biological roles of *circSTX6* in HCC, a variety of functional assays were performed. The efficiency of knockdown through transient and stable transfection was validated in MHCC97H, HCCLM3 and Huh7 cells using qPCR. The results confirmed that *circSTX6* was effectively silenced without affecting *STX6* expression, indicating precise targeting of siRNAs and shRNAs at the junction motifs of *circSTX6* (Figure 3A and Supplementary Figure S3A). As observed in the colony formation assay, CCK‐8 assay and flow cytometry, genetic ablation of *circSTX6* suppressed the proliferative capacities of tumour cells and intensified apoptosis (Figure 3B–F and Supplementary Figure S3B). What is more, EdU assays revealed a reduced DNA replication rate in HCC cells upon *circSTX6* knockdown (Figure 3G,H). To further evaluate the impact of *circSTX6* on tumourigenicity, subcutaneous implantation experiments were conducted. Significant slacked tumour growth along with reduced tumour volumes and weights were observed in *circSTX6*‐knockdown groups (*n* = 8) compared to that of the control group (*n* = 8) (Figure 3I,J). Moreover, the IHC staining of tumour markers, including Ki67, PCNA, E‐cadherin and N‐cadherin, indicated that the loss of *circSTX6* inhibited the proliferation and metastasis of HCC (Supplementary Figure S3C).

Conversely, a plasmid for *circSTX6* overexpression was successfully constructed and transfected into MHCC97H and HCCLM3 cells, which led to a significant increase in *circSTX6* expression, rather than *STX6*, as detected by qPCR (Supplementary Figure S4A). Colony formation and CCK‐8 assays revealed that the upregulation of *circSTX6* promoted the proliferation of HCC cells in vitro (Supplementary Figure S4B–D), and subcutaneous implantation experiments (*n* = 8 in each group) also showed accelerated tumour growth in vivo (Supplementary Figure S4E–G). Taken together, these findings demonstrate that *circSTX6* accelerates HCC proliferation both in vitro and in vivo.

### 3.4 *CircSTX6* promotes migration and invasion of HCC in vitro and in vivo

**FIGURE 4:**
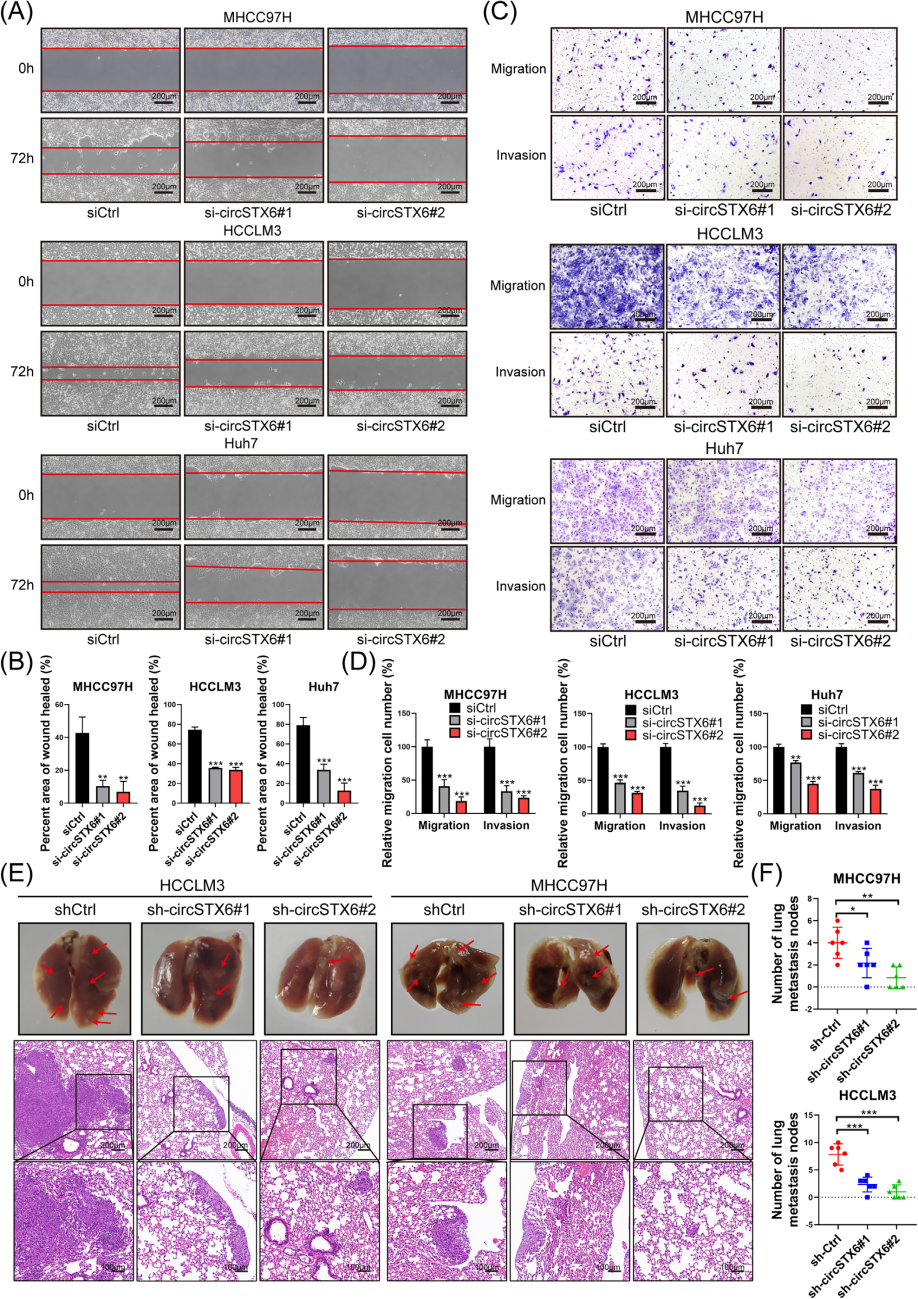
Ablation of *circSTX6* suppresses HCC migration and invasion in vitro and in vivo. (A) Migration capacity of HCC cells upon *circSTX6* knockdown was determined by wound‐healing assays in MHCC97H, HCCLM3 and Huh7 cells. Scale bar, 200 μm. (B) Percentages of wound‐healed areas are quantified in histograms. (C) Migration and invasion capabilities of MHCC97H, HCCLM3 and Huh7 were detected by transwell assays. Scale bar, 200 μm. (D) Relative count of migration and invasion cells is quantified in histograms. (E) Representative images and hematoxylin and eosin (HE) staining of metastatic nodules in different groups (*n* = 6). Scale bar, 100 μm. (F) Statistical analysis of metastatic pulmonary nodules in control and *circSTX6*‐knockdown models. *p < 0.05; **p < 0.01; ***p < 0.001; ns, not significant.

Wound healing and transwell assays were conducted to assess the effects of *circSTX6* on HCC cell migration. The results demonstrated that the downregulation of *circSTX6* restrained the migration capacities of MHCC97H, HCCLM3 and Huh7 cells (Figure 4A–C). In addition, transwell assays also indicated compromised invasiveness of HCC cells with *circSTX6* deficiency (Figure 4C,D). Next, to investigate the in vivo function of *circSTX6* in promoting migration and invasion, tail vein assays of tumour metastasis experiments were performed. The number of lung metastatic nodules (*n* = 6 in each group) was significantly reduced with the knockdown of *circSTX6*, and the results were confirmed by HE staining (Figure 4E,F). In contrast, overexpression of *circSTX6* notably promoted the migration and invasion of HCC cells, as demonstrated by wound‐healing and transwell assays (Supplementary Figure S4H–K). In conclusion, *circSTX6* promotes the migration and invasion of HCC both in vitro and in vivo.

### 3.5 *CircSTX6* directly interacts with HNRNPD protein in HCC cells

**FIGURE 5:**
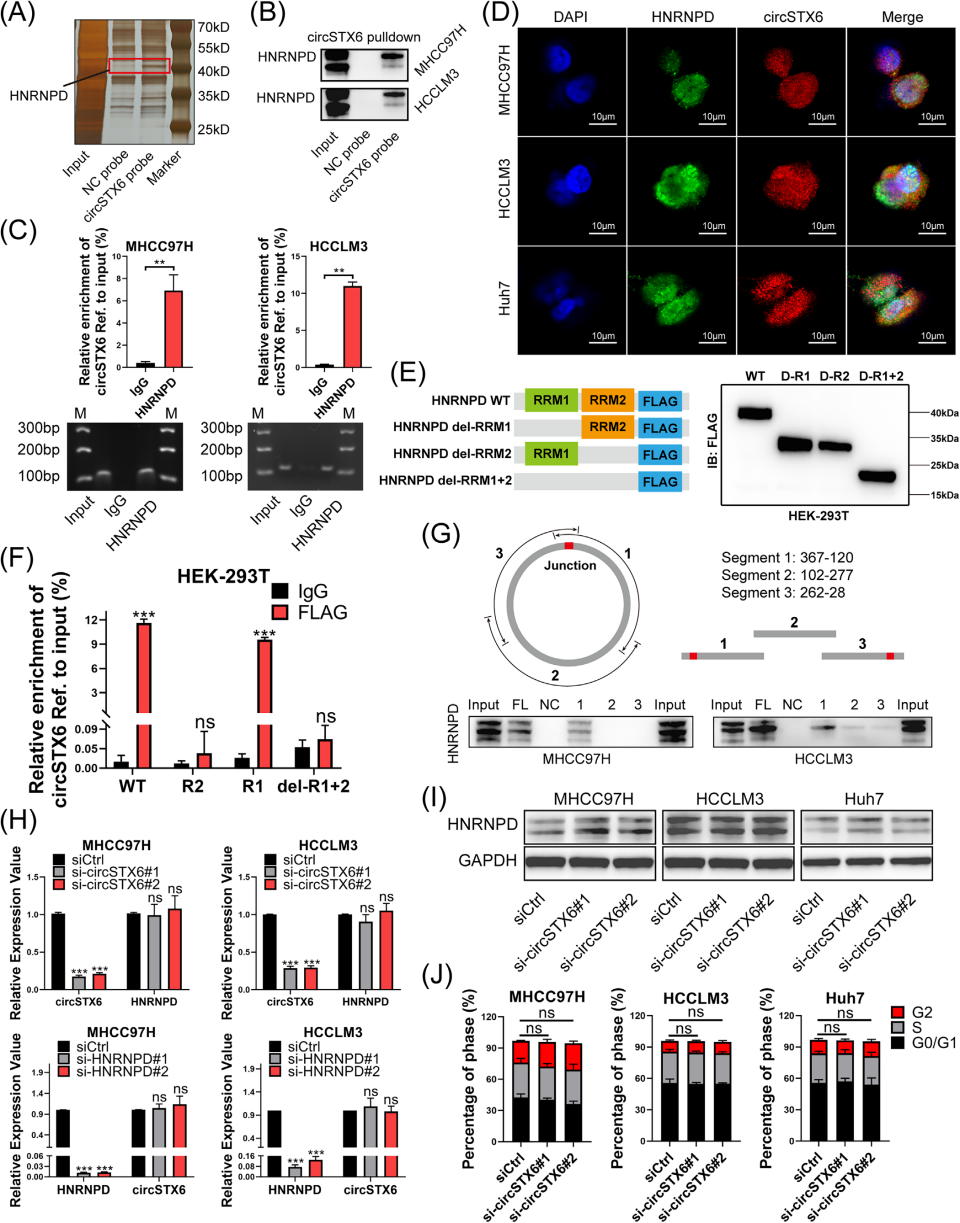
*CircSTX6* interacts with HNRNPD protein in HCC cells. (A) RNA pull‐down assay was performed in MHCC97H cells using biotinylated *circSTX6* and NC probes. Differential bands on the gel were visualized by silver staining and subsequently excised and sent for MS analysis. HNRNPD, with the largest unique peptide numbers, was chosen as the most potential binding protein of *circSTX6*. (B) The interaction of *circSTX6* and HNRNPD was confirmed by *circSTX6* pull‐down assay in HCC cells using Western blotting. (C) RIP assays followed by qPCR verified the enrichment of *circSTX6* by HNRNPD in HCC cells. The PCR products were subject to agarose gel electrophoresis. (D) FISH/IF assay showed co‐localization of *circSTX6* and HNRNPD proteins in the cytoplasm of HCC cells. Scale bar, 10 μm. (E) Plasmids of HNRNPD truncations (RRM1 and RRM2, labelled with a flag) were transfected into HEK‐293T cells, respectively. (F) *CircSTX6* was enriched using anti‐flag or anti‐IgG antibodies in each truncation group with RIP assay. The exogenous enrichment level of *circSTX6* was determined by qPCR. (G) The sequences of *circSTX6* were truncated into three segments. Specific probes targeting each segment were designed, and RNA pull‐down was performed in HCC cells utilizing full‐length (FL) probes or segment probes, respectively. (H) The expression of HNRNPD upon *circSTX6* knockdown and *circSTX6* upon HNRNPD knockdown was determined by qPCR in HCC cells, respectively. (I) The protein level of HNRNPD upon *circSTX6* knockdown was determined by Western blot. (J) Flow cytometry was conducted to determine the impact of *circSTX6* knockdown on the cell cycle in HCC cells. *p < 0.05; **p < 0.01; ***p < 0.001; ns, not significant.

Since previous studies have reported *circSTX6* as a miRNA sponge,^24^ we mainly focused on the *circSTX6*‐protein interaction in HCC cells in this study. RNA pull‐down assay was performed using biotinylated *circSTX6* probes to identify potential interacted proteins of *circSTX6*. Silver staining revealed a differential band at approximately 40 kDa between the negative control (NC) and *circSTX6* probe groups (Figure 5A). Mass spectrometry (MS) analysis identified HNRNPD as the protein with the highest Sequest Scores and most abundant unique peptides. Subsequent Western blot further confirmed the interaction between *circSTX6* and HNRNPD (Figure 5B). Consistently, the RIP assay verified that *circSTX6* could be enriched by anti‐HNRNPD antibody, as shown by qPCR results (Figure 5C). Besides, the FISH/IF assay also identified colocalization of HNRNPD and *circSTX6* in the cytoplasm of HCC cells (Figure 5D).

HNRNPD possesses two nonidentical RNA recognition motif (RRM) domains, which are crucial for high‐affinity RNA binding.^25^ To determine the precise domain responsible for RNA binding, HNRNPD was truncated and plasmids for flag‐tagged mutants were constructed. Western blotting verified the transfection efficiency of truncated proteins and their molecular weights (Figure 5E). Next, RIP assay was carried out and qPCR results showed that *circSTX6* could be efficiently enriched by wild‐type and RRM1 domain of HNRNPD but not the RRM2 (Figure 5F). Additionally, three biotinylated *circSTX6* segment probes were designed and the RNA pull‐down results indicated that *circSTX6* segment 1 was responsible for RNA binding with HNRNPD (Figure 5G). Collectively, these results suggest a direct interaction between HNRNPD and *circSTX6*.

Next, we examined whether *circSTX6* or HNRNPD affected the expression of each other. The qPCR and Western blot results demonstrated that *circSTX6* had little effect on the expression of HNRNPD at either the mRNA or protein level and vice versa (Supplementary Figure 5H,I and S5A). Interestingly, previous studies have reported that HNRNPD can promote the degradation of mRNAs such as *p21* (*CDKN1A*), *cyclin D1* (*CCND1*) and *p16* (*CDKN2A*), which are known to regulate the cell cycle.^26^, ^27^ Flow cytometry was performed to assess whether *circSTX6* influenced the cell cycle in an HNRNPD‐dependent manner. Notably, cell cycle regulation was not altered upon *circSTX6* silencing (Figure 5J). Therefore, it is highly conceivable that the other downstream targets might be regulated by *circSTX6* and HNRNPD.

### 3.6 *CircSTX6* promotes *ATF3* mRNA decay in an HNRNPD‐dependent manner

**FIGURE 6:**
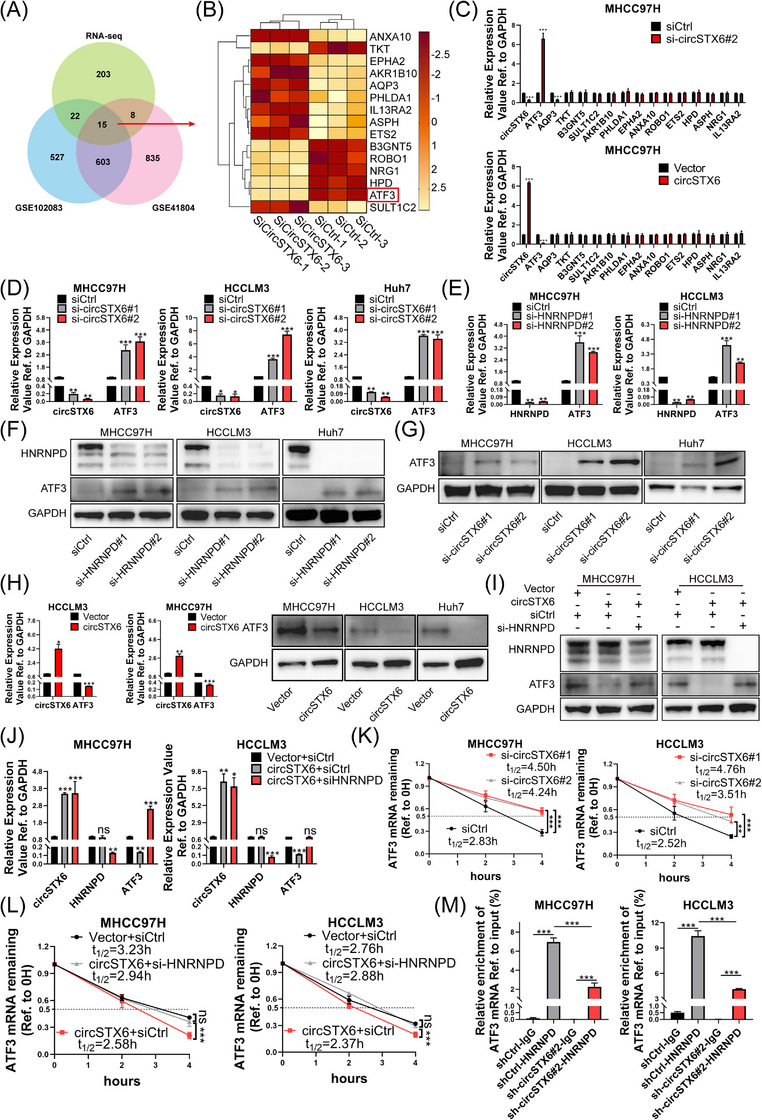
*CircSTX6* facilitates HNRNPD‐mediated *ATF3* mRNA decay. (A) Venn diagram showing the overlap of 15 candidate transcripts. RNA‐seq data were obtained by sequencing the MHCC97H cells (si‐*circSTX6*#2 vs. si‐Ctrl), including 248 differentially expressed genes (DEGs, average FPKM > 10, fold change > 1, *p* < 0.05). DEGs (fold change >1, *p* < 0.05) in GSE102083 and GSE41804 were obtained from sequencing data of HCC tumour tissues, containing 1167 and 1461 transcripts, respectively. (B) Clustered heatmap presenting the expression of 15 genes between groups of si‐*circSTX6*#2 and si‐Ctrl MHCC97H cells. White and red bars indicated upregulation and downregulation, respectively. (C) The expression of 15 genes was preliminarily examined by qPCR, and only *ATF3* was stably regulated by *circSTX6* knockdown or overexpression. (D) The expression of *ATF3* was examined by qPCR in *circSTX6*‐silenced MHCC97H, HCCLM3 and Huh7 cells. (E) The expression of *ATF3* was examined by qPCR in HNRNPD‐silenced HCC cells. (F and G) The expression of ATF3 was examined by Western blotting in HNRNPD‐ or *circSTX6*‐silenced HCC cells. (H) The expression of ATF3 upon *circSTX6* overexpression was examined by qPCR and Western blotting. (I and J) The expression of *circSTX6*, ATF3 and HNRNPD were detected by Western blotting and qPCR assays in *circSTX6*/HNRNPD rescue groups. (K) Relative RNA levels of *ATF3* were detected by qPCR at 0, 2 and 4 h in circSTX6‐knockdown or control HCC cells treated with actinomycin D (10 μg/mL). (L) RNA levels of *ATF3* were detected by qPCR at 0, 2 and 4 h after actinomycin D (10 μg/mL) treatment in *circSTX6* overexpression, *circSTX6* overexpression in combination with HNRNPD silencing and negative control HCC cell groups. (M) HNRNPD‐RIP assay was performed in *circSTX6*‐silenced or negative control HCC cells, followed by qPCR to detect the expression of *ATF3* mRNA and to assess the impact of *circSTX6* on the affinity of HNRNPD protein binding to *ATF3* mRNA. *p < 0.05; **p < 0.01; ***p < 0.001; ns, not significant.

To identify potential targets regulated by *circSTX6* and HNRNPD, RNA‐seq was performed with *circSTX6*‐ knockdown and negative control MHCC97H cells. As expected, Kyoto Encyclopedia of Genes and Genomes (KEGG) pathway analyses showed significant enrichment in pathways related to cancer, focal adhesion, Phosphatidylinositol 3‐kinase (PI3K)‐Akt kinase (AKT) signalling and Mitogen‐activated protein kinase (MAPK) signalling (Supplementary Figure S5B). Next, mRNA expression profiles from HCC tumour tissues based on the Gene Expression Omnibus database were employed (Figure 6A). A total of 618 differentially expressed genes (DEGs) were obtained from the intersection of two datasets (GSE102083 and GSE41804) (Figure 6A), and 15 candidate genes were subsequently screened out (Figure 6B). The expression of these candidate genes was examined by qPCR in *circSTX6* knockdown or overexpressing HCC cells. *ATF3* was identified as a potential downstream target of circSTX6, as its expression was consistently upregulated upon *circSTX6* silencing, and downregulated upon *circSTX6* overexpression (Figure 6C and Supplementary Figure S5C). The expression of *ATF3* was further checked using two siRNAs of *circSTX6* in HCC cells (Figure 6D), and the result was consistent. Thus, we identified *ATF3* as a potential downstream target of *circSTX6*.

Next, we sought to determine the relationship between HNRNPD and *ATF3*. The qPCR and Western blotting assays showed that knockdown of either *circSTX6* or HNRNPD increased the expression of *ATF3* at both mRNA and protein levels (Figure 6E–G). Moreover, overexpressing *circSTX6* in HCC cells reduced *ATF3* expression (Figure 6H and Supplementary Figure S5D). These results suggested that *circSTX6* and HNRNPD work synergistically to regulate *ATF3*. Intriguingly, a previous study showed that HNRNPD could promote the decay of *ATF3* mRNA by binding to it.^28^ Therefore, we wondered whether *circSTX6* influences *ATF3* expression by facilitating the HNRNPD‐mediated *ATF3* mRNA degradation and thus drives HCC malignancies. To verify this speculation, we first performed Western blot and qPCR. Results showed that the knockdown of HNRNPD retrieved the downregulation of *ATF3* triggered by *circSTX6* overexpression (Figure 6I,J), indicating that *circSTX6* regulates *ATF3* expression in an HNRNPD‐dependent manner. Moreover, silencing of *circSTX6* also rescued the downregulation of ATF3 resulting from HNRNPD overexpression (Supplementary Figure S5E), indicating the indispensable role of *circSTX6* in the degradation of *ATF3* by HNRNPD. Next, we conducted RNA stability assays to assess the mRNA decaying rate of *ATF3* upon *circSTX6* silencing. Consistently, inhibition of *circSTX6* contributed to a longer half‐life of *ATF3* mRNA (Figure 6K). In contrast, overexpression of *circSTX6* reduced the stability of *ATF3* mRNA and led to a decreased half‐life, while this effect was abolished by HNRNPD knockdown (Figure 6L). Subsequently, an RIP assay was performed to identify the binding of HNRNPD with *ATF3* mRNA (Figure 6M). Notably, the loss of *circSTX6* attenuated HNRNPD binding to *ATF3* mRNA (Figure 6M), indicating that the conjugation of *circSTX6* and HNRNPD increased the binding affinity of HNRNPD to *ATF3* mRNA. Conversely, overexpression of *circSTX6* increased the binding of *ATF3* mRNA to HNRNPD (Supplementary Figure S5F). Collectively, *circSTX6* sponges HNRNPD to facilitate its binding with *ATF3* mRNA, thus reducing *ATF3* mRNA stability.

Additionally, we examined the presence of other potential targets coregulated by HNRNPD and *circSTX6* that play a part in HCC pathogenesis. The expression of 10 potential targets (previously reported targets of HNRNPD) was examined by qPCR with *circSTX6* silencing or overexpression (Supplementary Figure S5G,H).^26^, ^29^, ^30^, ^31^ Notably, *Interleukin 8* (*IL8*) was found to be stably regulated by *circSTX6*, and it has been established as an important tumourigenic factor in HCC.^32^, ^33^ Our findings suggest that the interaction of *circSTX6* and HNRNPD promotes HCC progression through diverse pathways, and *ATF3* represents only one of the downstream targets. Nevertheless, we mainly focused on the *circSTX6*‐HNRNPD‐*ATF3* axis in this study.

### 3.7 Dysregulated *circSTX6*‐*ATF3* axis drives HCC malignancies

**FIGURE 7:**
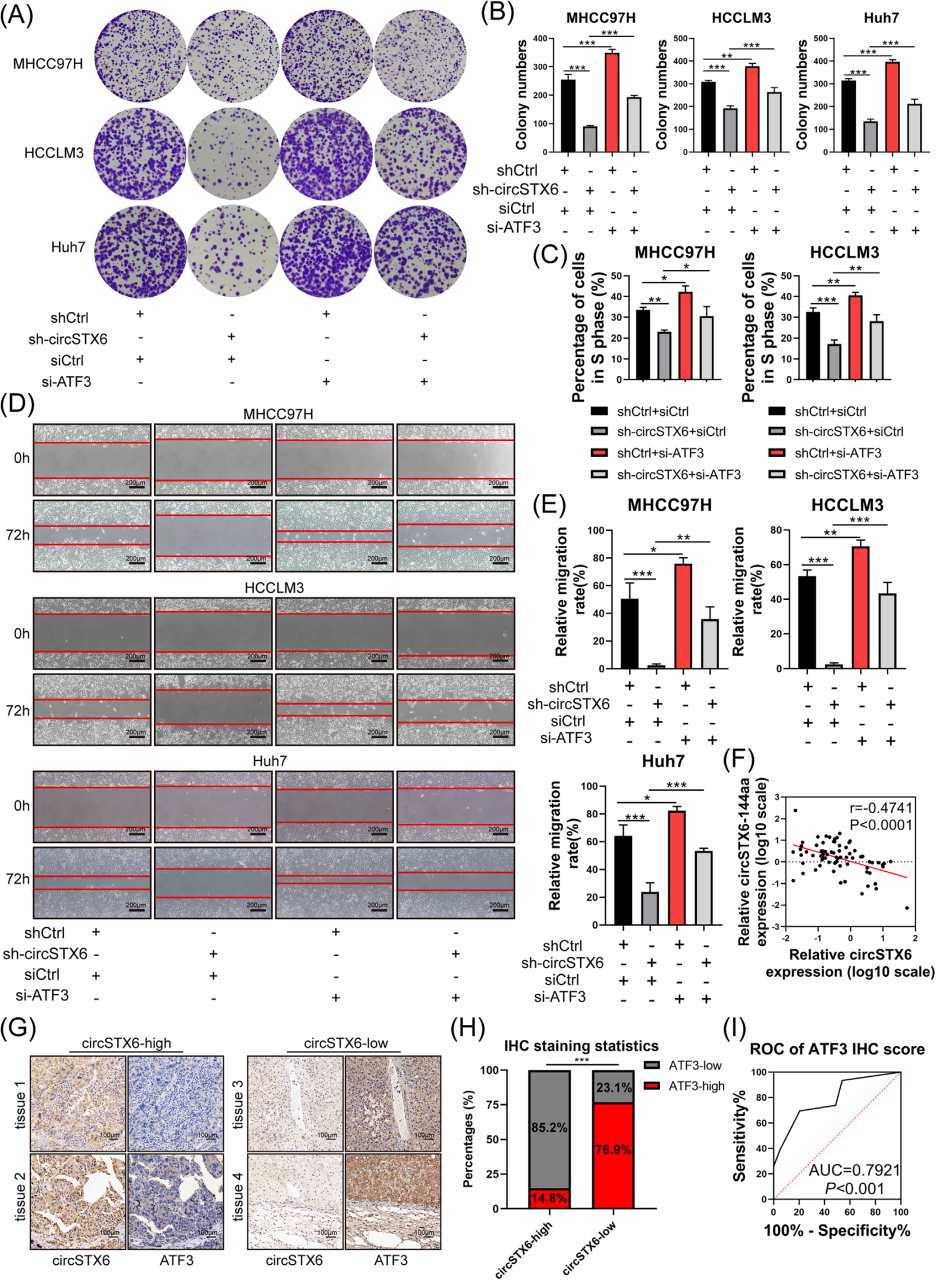
Inhibition of *ATF3* rescues the effects of *circSTX6* knockdown. (A) Colony formation assay was performed to assess the proliferation of MHCC97H, HCCLM3 and Huh7 cells with either *ATF3* inhibition or *circSTX6* knockdown. (B) The colony numbers of the colony formation assay were quantified in histograms. (C) Statistical analysis of percentages of cells in the S phase in EdU assays. (D) Wound‐healing assay was employed in either *circSTX6* silencing or *ATF3*‐deficient HCC cells. Scale bar, 200 μm. (E) Relative migration rate of HCC cells is exhibited. (F) Expression of *ATF3* and *circSTX6* in HCC cohort‐1 (*n* = 72) was detected by qPCR. Their expression was analysed with Pearson correlation analysis. (G) IHC staining of ATF3 and ISH staining of *circSTX6* were respectively performed based on HCC cohort‐2 (*n* = 40). Representative images of *cricSTX6* with varying degrees of dyeing intensity and corresponding ATF3 images in the same sample are shown. Scale bar, 100 μm. (H) The percentages of high‐ or low‐expression *circSTX6* or ATF3 in HCC specimens are displayed in histograms. (I) Diagnostic ROC curve based on the IHC score of ATF3 in HCC cohort‐2 (AUC = 0.7921, *p* < 0.001). *p < 0.05; **p < 0.01; ***p < 0.001; ns, not significant.

To determine whether *circSTX6* drives HCC tumourigenesis and progression in an ATF3‐mediated manner, a series of functional rescue assays were conducted. Colony formation and CCK‐8 assays revealed that the loss of *circSTX6* reduced the proliferation capacities of HCC cells, while silencing of *ATF3* reversed this phenomenon (Figure 7A,B and Supplementary Figure S6A). Similarly, phenotypes of the EdU assay were rescued (Figure 7C and Supplementary Figure S6B). Furthermore, the inhibition of *ATF3* partially attenuated the impaired capabilities of invasion and migration in HCC cells caused by *circSTX6* knockdown (Figure 7D,E and Supplementary Figure S7A–D). In sum, dysregulated *ATF3* might account for the *circSTX6*‐driven malignant behaviours of HCC.

Additionally, the clinical correlation of the *circSTX6*‐*ATF3* axis was assessed. The expression of *circSTX6* and *ATF3* in HCC cohort‐1 was determined by qPCR. Pearson correlation analyses showed a negative correlation between *circSTX6* and *ATF3* (*r* = −0.4741, *p* < 0.0001; Figure 7F). Moreover, we examined the expression of *ATF3* in the HCC cohort based on the TCGA database (*n* = 369) and *ATF3* was significantly downregulated in tumour tissues compared with adjacent tissues (Supplementary Figure S7E). Besides, the costaining of *circSTX6* and ATF3 in HCC cohort‐2 revealed that approximately 85.2% of the *circSTX6* highly expressed tumour tissues weakly expressed ATF3, whereas 76.9% of *circSTX6* weakly expressed tumour tissues showed stronger ATF3 staining (Figure 7G,H). Consistently, ROC curves showed that the AUC value of ATF3 was 0.7921 (*p* < 0.001; Figure 7I), suggesting ATF3 could be used to distinguish between HCC tumour and normal tissues. Therefore, the expression of *circSTX6* and ATF3 is negatively correlated in HCC and the *circSTX6*‐*ATF3* axis could be a promising therapeutic target.

### 3.8 *CircSTX6* encodes a novel 144aa protein, circSTX6‐144aa, that serves a good prognostic biomarker

**FIGURE 8:**
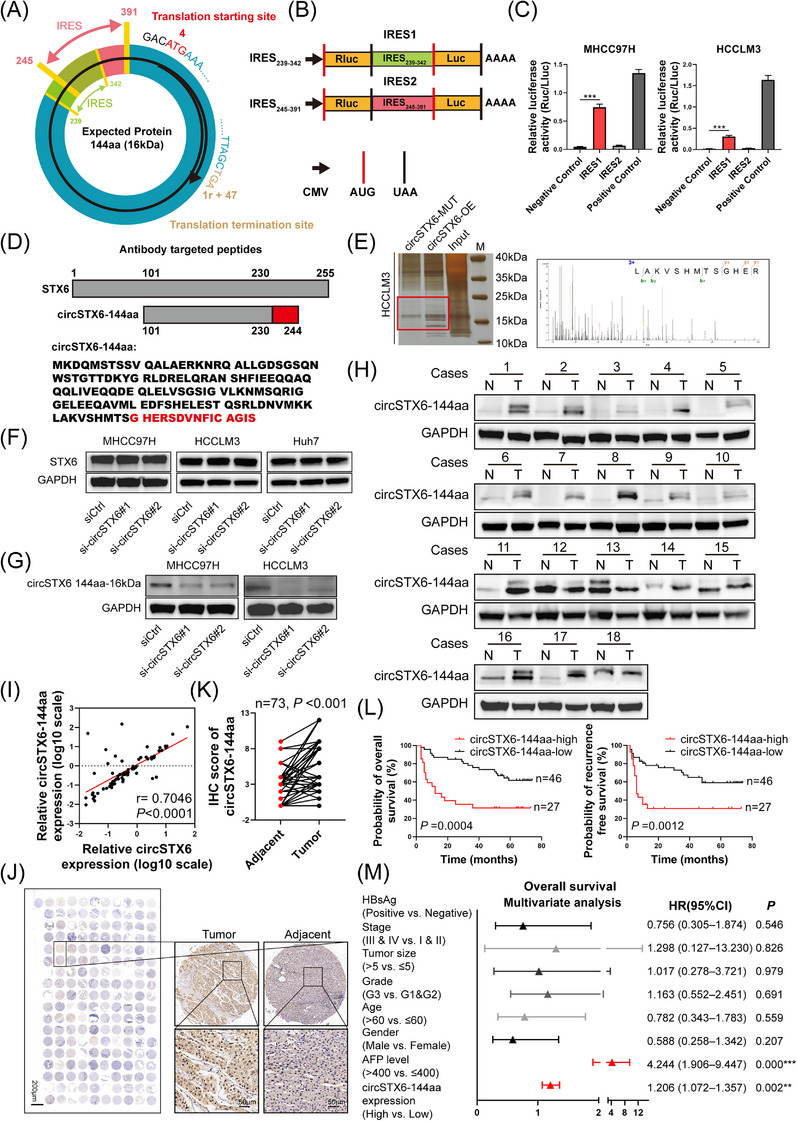
CircSTX6‐144aa is a novel protein encoded by *circSTX6*. (A) Schematic illustration of potential IRES sequences (at 245–391 and 239–342 nt, respectively) and ORF sequences on *circSTX6*. (B) Schematic illustration of the construction of dual luciferase reporter plasmids. IRES sequences were cloned between the *Renilla* and firefly *luciferase* genes with AUG and UGA codons. (C) Luciferase activity was detected in HCC cells transfected with IRES1 or IRES2 plasmids, respectively. (D) Schematic illustration of STX6 and circSTX6‐144aa peptide sequences. The unique peptides of circSTX6‐144aa were marked red. (E) HCCLM3 cells were transfected with *circSTX6* or *circSTX6* with ATG mutant plasmids, respectively; silver staining showed differential bands, and the gel was excised and sent for MS analysis. The identified polypeptide contained unique sequences of circSTX6‐144aa. (F) The expression of STX6 in *circSTX6*‐knockdown or negative control HCC cells. (G) Expression of circSTX6‐144aa in *circSTX6* knockdown or negative control HCC cells. (H) Expression of circSTX6‐144aa was detected in 18 pairs of HCC tumour and adjacent normal tissues. (I) Expression of circSTX6‐144aa in HCC cohort‐1 was determined by qPCR (*n* = 72). Correlation analyses between circSTX6‐144aa and *circSTX6* were performed. (J) The overall and representative images of IHC staining of circSTX6‐144aa. Scale bar in the overall image, 200 μm; scale bar in representative images, 50 μm. (K) IHC scores in 73 pairs (HCC cohort‐3) of HCC tumour and adjacent tissues were compared. (L) Kaplan–Meier prognoses analysis showing the OS (*n* = 73) and RFS (*n* = 73) of HCC patients based on circSTX6‐144aa expression. (M) Forest plot shows the hazard ratio (HR) and 95% confidence intervals (CI) of risk factors in HCC cohort‐3 based on Cox multivariate analysis. High expression of circSTX6‐144aa and AFP levels were independent risk factors. *p < 0.05; **p < 0.01; ***p < 0.001; ns, not significant.

Furthermore, we sought to determine the protein translation capacity of *circSTX6*. As predicted by circRNADb software,^34^ *circSTX6* has two potential internal ribosome entry sites (IRES) at 245–391 nt and 239–342 nt, respectively, and one open reading frame (ORF) that encodes a putative 144aa protein (Figure 8A and Supplementary Figure S8A). Moreover, the start codon ATG and ORF sequences formed by *circSTX6* back splicing were verified in ORF Finder (https://www.ncbi.nlm.nih.gov/orfnder/) (Supplementary Figure S8B).^35^ Herein, we termed the 144aa novel protein as circSTX6‐144aa. To determine which IRES drove the translation of *circSTX6*, dual‐luciferase assays were performed (Figure 8B), which demonstrated that IRES1 (245–391 nt) significantly increased the luciferase activity compared with IRES2 (239–342 nt) (Figure 8C). Driven by IRES1 (245–391 nt), circSTX6‐144aa shares the same sequence with wild‐type STX6 at 101−230 and contains unique sequences at the C‐terminal (Figure 8D). Next, to confirm the existence of circSTX6‐144aa, we transferred the *circSTX6* overexpression plasmid and *circSTX6* ATG mutant plasmid (*circSTX6*‐MUT) into HCCLM3 cells. We performed silver staining, and the results showed a distinct band at approximately 16 kDa, consistent with the predicted molecular weight (Figure 8E). Then the target band was excised and sent for MS analysis. MS analysis detected the unique GHER sequence.

To specifically detect circSTX6‐144aa, we constructed a custom‐designed antibody targeting partial sequences of circSTX6‐144aa (Supplementary Table S6). Western blotting was conducted to examine the presence of translated endogenous circSTX6‐144aa. Notably, upon *circSTX6* silencing, the expression of the parental gene *STX6* was not altered (Figure 8F), whereas circSTX6‐144aa was significantly reduced in MHCC97H, HCCLM3 and Huh7 cells (Figure 8G and Supplementary Figure S8C). In addition, we constructed a linear circSTX6‐144aa overexpression plasmid. Western blot showed overexpressed circSTX6‐144aa in *circSTX6*‐OE and linear circSTX6‐144aa groups, rather than the *circSTX6*‐MUT group (Supplementary Figure S8D), indicating antibody specificity. Next, we sought to identify the expression of circSTX6‐144aa in HCC tumour tissues. Interestingly, Western blotting showed that circSTX6‐144aa was overexpressed in 32/38 (84.21%) tumour tissues compared to that of adjacent tissues (Figure 8H and Supplementary Figure S8E). Moreover, circSTX6‐144aa was positively correlated with the expression of *circSTX6* in 72 pairs of HCC tumour tissues according to qPCR (Figure 8I). To evaluate the clinical relevance of circSTX6‐144aa, we performed IHC staining using the custom‐designed antibody in HCC cohort‐3 (Figure 8J). Consistently, overexpression of circSTX6‐144aa was observed in HCC tumour tissues (Figure 8K) and its upregulation was associated with a worse overall prognosis (Figure 8L). Furthermore, the expression of circSTX6‐144aa was considered an independent risk factor for OS of HCC patients (Figure 8 M). Taken together, these results indicate that *circSTX6*‐translated 144aa could be a potential biomarker for HCC.

### 3.9 CircSTX6‐144aa promotes HCC proliferation, migration and invasion independent of *circSTX6*

**FIGURE 9:**
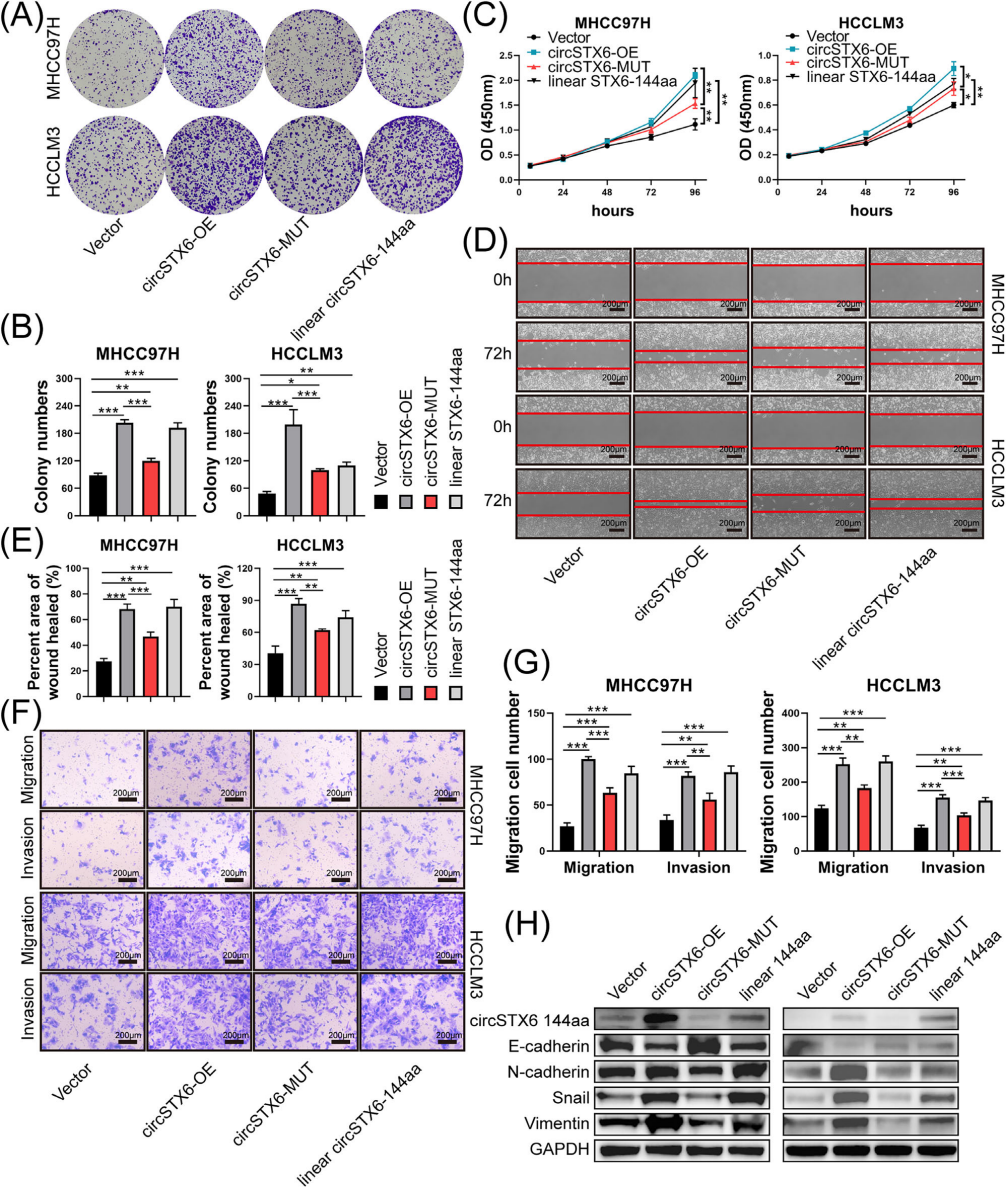
CircSTX6‐144aa promotes HCC proliferation, migration and invasion independent of *circSTX6*. (A) Colony formation assay to evaluate the proliferation capacity of HCC cells with overexpression of *circSTX6*, *circSTX6* with ATG mutant and linear circSTX6‐144aa. (B) Colony numbers are demonstrated in histograms. (C) CCK‐8 assay to evaluate the effect of circSTX6‐144aa on the proliferation of HCC cells. (D–G) Wound healing and transwell assays to evaluate the effect of circSTX6‐144aa on migration and invasion of HCC cells. The percentage area of wound healed and migration cell numbers are demonstrated by histograms. (H) The protein levels of N‐cadherin, E‐cadherin, Snail and Vimentin in HCCLM3 and MHCC97H cells were detected by Western blot. *p < 0.05; **p < 0.01; ***p < 0.001; ns, not significant.

**FIGURE 10:**
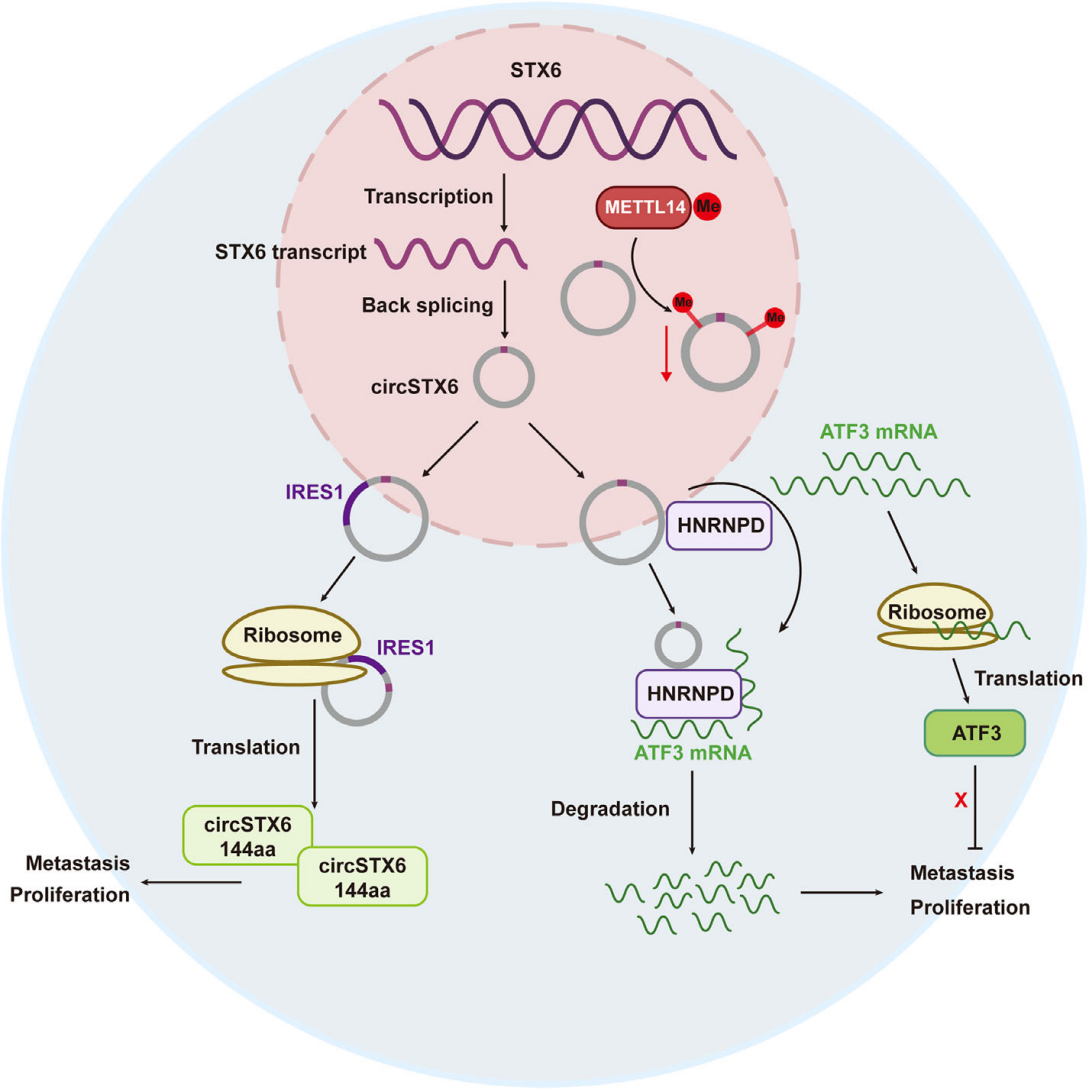
Proposed model illustrating the underlying mechanisms of *circSTX6* in HCC. METTL14‐mediated m^6^A modification on *circSTX6* inhibits its expression. *CircSTX6* promotes the proliferation and metastasis of HCC by binding to HNRNPD to facilitate HNRNPD‐mediated *ATF3* mRNA degradation. Driven by IRES1, *circSTX6* encoded a 144 amino acid novel polypeptide, circSTX6‐144aa, which promotes HCC progression independent of *circSTX6* itself.

To further confirm the biological functions of circSTX6‐144aa and verify whether these effects are independent of *circSTX6* itself, a series of rescue assays were conducted. As expected, overexpression of either *circSTX6* or linear circSTX6‐144aa significantly promoted the proliferation of HCC cells by colony formation and CCK‐8 assays (Figure 9A–C) as well as migration and invasion by wound‐healing and transwell assays (Figure 9D–G). Notably, the overexpression of *circSTX6* with ATG mutant, which abrogates the translation of circSTX6‐144aa, could also increase the proliferation, migration and invasion capacity of HCC cells (Figure 9A–G), indicating that circSTX6‐144aa promoted HCC malignancies independent of *circSTX6* itself. Furthermore, we examined whether circSTX6‐144aa enhanced the migration and invasion of HCC cells through epithelial‐mesenchymal transition. The Western blot results showed that the expression of Vimentin, Snail and N‐cadherin increased upon circSTX6‐144aa overexpression, but E‐cadherin decreased (Figure 9H). These results reveal that both *circSTX6* and circSTX6‐144aa play an oncogenic role in HCC. Based on our results, the potential mechanisms are illustrated in Figure 10.

## 4 DISCUSSION

CircRNAs are involved in tumour biology by acting as miRNA sponges, serving as protein scaffolds and encoding proteins, in which miRNA sponge is most widely reported. Significantly, mounting research is paying attention to the other two mechanisms, especially protein translation, which can be driven by IRES or m^6^A modification on circRNAs.^20^ In our current study, using genome‐wide mapping of m^6^A‐immunoprecipitated circRNAs, we identified *circSTX6* which, by coincidentally, has been reported to function as a miRNA sponge to promote the progression of pancreatic adenocarcinoma.^24^ However, the full range of functions of *circSTX6* in HCC beyond miRNA sponge is still unclear. Our results confirmed an increased expression of *circSTX6* in HCC, and its high expression was associated with advanced tumour stages, higher pathological grades and worse prognoses. In addition, loss‐ and gain‐of‐function assays demonstrated that *circSTX6* played an oncogenic role by enhancing cell proliferation, migration and invasion capabilities in vitro and in vivo in HCC. Thus, *circSTX6* could be a candidate prognostic and therapeutic target in HCC.

Over the years, the interplay between m^6^A modification and ncRNAs, especially circRNAs, has gained increasing attraction.^36^ METTL14, an auxiliary activator of METTL3, although less reported, has been shown to participate in circRNA regulation through m^6^A‐dependent manners.^14^ To be exact, Fan et al. revealed that METTL14‐mediated m^6^A modification on *circORC5* decreases its expression and inhibits gastric cancer development.^19^ Consistently, our results showed that *circSTX6* expression was regulated by METTL14 in an m^6^A‐dependent manner and such regulation also existed in HCC tumour and adjacent normal tissues, which extended our knowledge of m^6^A‐modified circRNAs in tumour progression. Nevertheless, m^6^A modification is a complex biological process that requires coordinated operations of many regulators, including ‘writers’ of METTL3, WTAP, ‘readers’ of IGF2BPs and YTHDFs and ‘erasers’ of ALKBH5 and FTO, and our study only focused on the interaction between METTL14 and *circSTX6*. It remains unclear whether other regulators (such as METTL3 and FTO) that catalyze methylation/demethylations on m^6^A sites regulate circSTX6 in different manners (such as cytoplasmic export or splicing). Besides, emerging evidence has proved the encoding capacity of circRNAs, which could be driven by m^6^A in a variety of malignancies.^11^, ^37^, ^38^ For instance, c*ircMAP3K4* encodes a 455aa polypeptide by means of IGF2BP1‐mediated m^6^A modification and promotes HCC progression.^17^ In this study, we established that *circSTX6* could be translated into 144aa polypeptide, circSTX6‐144aa and it possesses good performance in prognostic prediction. In addition, circSTX6‐144aa could promote tumour progression independent of *circSTX6* itself, serving an oncogenic role in HCC. Nevertheless, current evidence only supports the initiation of *circSTX6* translation by IRES (Figure 8C) instead of m^6^A modification (data not provided) and the detailed mechanisms underlying circSTX6‐144aa and m^6^A warrant further exploration.

The functional role of circRNAs is closely associated with their subcellular localization.^39^ For example, circRNAs located in the nucleus are usually involved in transcriptional regulation or splicing events^40^, ^41^ whereas cytoplasmic circRNAs frequently serve as miRNA sponges or protein scaffolds.^24^, ^42^ In the current study, *circSTX6* was predominantly located in the cytoplasm, consistent with the previous research. Therefore, using circRNA pulldown, MS analysis, RIP and FISH/IF, we identified HNRNPD as a robust interactor with *circSTX6*. HNRNPD, also known as adenylate‐uridylate‐rich element/poly(U)‐binding/degradation factor 1 (AUF1), encompasses four isoforms generated by alternative splicing of a precursor mRNA (pre‐mRNA).^25^, ^29^ The four protein isoforms (p37, p40, p42 and p45) of HNRNPD all share several common structural elements that include two non‐identical RRM domains, two tandems and eight amino acid glutamine‐rich sequences.^25^, ^43^ Nevertheless, there is no consensus about the precise subcellular localization of each isoform. This and the lack of HNRNPD isoform‐specific antibodies make it difficult to determine which isoform associates with circSTX6 in detail.^44^ Since previous studies have revealed that the two RRM domains are necessary for high‐affinity RNA binding, we constructed corresponding truncation mutants.^45^ The RIP assay confirmed that the RRM1 domain of HNRNPD mediated its binding to *circSTX6*. Conversely, the precise RNA sequences required for binding were also truncated and the RNA pull‐down revealed that truncation 1 showed the highest affinity. Hence, our study provided compelling evidence that *circSTX6* serves as a scaffold for HNRNPD, elucidated their interaction and broadened the understanding of binding motifs on circRNAs. However, it is worth noting that more stringent experiments considering circRNA secondary structure and protein conformation are warranted in future exploration.

ATF3 is a stress‐induced transcription factor that belongs to the ATF/cyclin adenosine monophosphate response element‐binding protein family.^46^ It has been reported to serve as a tumour suppressor in HCC. Chen and his colleagues revealed that ATF3 inhibited HCC progression through the transcriptional regulation of cysteine‐rich angiogenic inducer 61 (CYR61).^47^ Notably, ATF3 can be regulated by ncRNAs through diverse mechanisms, such as miRNA‐mediated transcriptional inhibition and circRNA‐guided competing endogenous RNA patterns.^48^, ^49^ Although the interaction between ATF3 and circRNAs has been explored in a few studies, the underlying post‐transcriptional mechanisms remain elusive. A canonical but crucial function of HNRNPD is controlling the mRNA decay.^50^ Notably, Wang et al. found that HNRNPD could antagonize ferroptosis by destabilizing the *ATF3* mRNA.^51^ Mechanistically, HNRNPD promoted *ATF3* mRNA decay by directly binding to its 3′‐untranslated region. Furthermore, Pan et al. also established that HNRNPD regulated *ATF3* expression under stress conditions in the HCC cell line HepG2.^28^ The RIP assay by Pan and his colleagues demonstrated decreased HNRNPD binding to *ATF3* mRNA along with elevated *ATF3* expression and declined mRNA decaying. Being consistent with previous studies, our research showed that *circSTX6* served as an enhancer for HNRNPD‐mediated *ATF3* mRNA decay based on mRNA stability assays. Silencing of *circSTX6* slowed the degradation of *ATF3* mRNA. Moreover, the RIP rescue assay indicated attenuated HNRNPD binding to *ATF3* mRNA upon circSTX6 knockdown. Overall, we enriched the current understanding of *ATF3* metabolism through circRNA‐RBP sponge mechanisms.

Nevertheless, there are still several limitations in this work that require further investigation. First, whether the biogenesis (backsplicing) or translocation of *circSTX6* is controlled by m^6^A and in which way remains largely unknown. Additionally, we only validated one essential target (*ATF3*) of *circSTX6* and HNRNPD, while other potential targets (such as *IL8*) exist and also take part in the progression of HCC. Furthermore, the oncogenic mechanisms of circSTX6‐144aa are not fully revealed compared to *circSTX6* itself.

## 5 CONCLUSION

In conclusion, our study revealed the crucial roles of *circSTX6* in HCC tumourigenesis and progression. METTL14 regulated *circSTX6* expression in an m^6^A‐dependent manner. *CircSTX6* enhanced HNRNPD‐mediated *ATF3* mRNA degradation, thereby promoting HCC growth and metastasis. Additionally, *circSTX6* could be translated into a 144aa polypeptide, circSTX6‐144aa, that served as a good biomarker and promoted HCC progression independent of *circSTX6* itself. Clinically, *circSTX6* was significantly upregulated in HCC tissues and high *circSTX6* expression indicated an unfavourable prognosis for HCC patients. In addition, the expression of *circSTX6* in tumour tissues was negatively associated with METTL14 and ATF3 but positively correlated with circSTX6‐144aa. Taken together, our study illustrated that *circSTX6* and its encoded circSTX6‐144aa are potential biomarkers for HCC diagnosis and prognosis. Targeting *circSTX6* could be an effective strategy for HCC treatment.

## CONFLICT OF INTEREST STATEMENT

The authors declare that they have no competing interests.

## Supporting information

Supporting information

## Data Availability

The authors declare that all the original data of this study are available within the article or under reasonable request from the corresponding authors.
