# Supplementary material for: N 6‐methyladenosine‐modified circSTX6 promotes hepatocellular carcinoma progression by regulating the HNRNPD/ATF3 axis and encoding a 144 amino acid polypeptide
Source: Clin Transl Med. 2023 Oct 25;13(10):e1451. doi: 10.1002/ctm2.1451 (PMC10599281; doi:10.1002/ctm2.1451)
Supplement: Supplementary file 1 — Supporting information [file CTM2-13-e1451-s001.docx]

**Supplementary Materials and Methods, Supplementary Figures S1-S8 and Supplementary Tables S1-S6.**

**N6-methyladenosine-modified *circSTX6* promotes hepatocellular carcinoma progression by regulating the HNRNPD/*ATF3* axis and encoding a 144 amino acid polypeptide**

**This file includes:**

Supplementary Materials and Methods

Supplementary Figures S1-S8 and corresponding figure legends

Supplementary Tables S1-S6

**Supplementary Materials and Methods**

**RNase R digestion assay**

The total RNA extracted from MHCC97H, HCCLM3 and Huh7 cells were equally divided. One group was treated with RNase R (Epicentre, USA) at 37℃ for 30 min, with a ratio of 3U for 1 μg RNA. The other group served as control. The expression of *circSTX6* and *STX6* were determined in each group by RT-qPCR. *GAPDH* in the control group served as the internal reference.

**Actinomycin D treatment and RNA stability assay**

MHCC97H, HCCLM3 and Huh7 cells reached 80% confluence (5×10^5^ cells) in the 6-well plate were treated with Actinomycin D (SBR00013, Sigma Aldrich) with a concentration of 10 μg/mL. And the cells were harvested at the indicated time points, respectively. Then qPCR was performed and the RNA degradation rate was calculated and normalized based on 0 h.

**RNA fractionation assays**

A total of 1×10^6^ cells were used for the fractionation of cytoplasmic and nuclear RNA. All procedures were in accordance with the manuals by the Cytoplasmic and Nuclear RNA Purification Kit (Norgen, Canada). The *GAPDH* and *U6* served as internal references in cytoplasm or nuclear, respectively.

**Methylated RNA immunoprecipitation (MeRIP)**

The MeRIP assay was performed with the EpiQuik CUT&RUN m^6^A RNA Enrichment (MeRIP) Kit (P-9018-24, Epigentek) according to manufacturer’s guidelines. In brief, 20 μg (protocol recommendation) of total RNA was isolated from HCC cells and mixed with 2 μg m^6^A antibodies along with affinity beads. They were incubated at room temperature for 90 minutes for the immunocapture of RNA fragments. Next, the nuclear digestion enhancer and cleavage enzyme mix were supplemented for RNA cleavage. Consequently, the m^6^A-labeled RNA fragments were captured by RNA binding beads, eluted and recovered. Specific primers targeting the m^6^A sites were designed and listed in Supplementary Table S5. The enrichment fold was normalized to the input.

**Sanger sequencing**

PCR product from *circSTX6* divergent primers were subject to Sanger sequencing by Tsingke Biological Technology (Beijing, China).

**ISH, IHC, histology and Western blotting assays**

ISH assay was conducted based on HCC cohort-2 and cohort-3 using a digoxin-labeled probe targeting *circSTX6* junction sites. The slides were de-paraffinized and pretreated with proteinase K (20μg/mL), followed by blocking with the 3% H_2_O_2_. Then the slides were prehybridized at 37℃ for 1 h and hybridized with *circSTX6* probe (5ng/μL) at 37℃ overnight. At last, DAB and haematoxylin were dyed targeting *circSTX6* and nuclei respectively, and visualized by CaseViewer (3DHISTECH Ltd., Hungary). The probe sequences for *circSTX6* were listed as follows: hsa_circ_0007905-Dig-1: Digoxin-5’-TCTGATCTTTCATGTCCACTGGTCATATGA-3’-Digoxin; hsa_circ_0007905-Dig-2: Digoxin-5’-ATCTTTCATGTCCACTGGTCAT-3’-Digoxin.

The subcutaneous tumor specimens and lungs with pulmonary metastasis from mice were fixed with 4% paraformaldehyde and embedded in paraffin, followed by IHC staining (Ki67, PCNA, E-cadherin and N-cadherin) and HE staining respectively. In addition, IHC staining of METTL14 and ATF3 were conducted on HCC cohort-2, while circSTX6-144aa on HCC cohort-3. All antibodies were supplemented in Supplementary Table S6.

Staining scores of ISH/IHC were assessed independently by two ten-year-experience pathologists based on the staining intensity (degree of 0, 1, 2, 3 represented negative, weak-, moderate-, and strong-positive, respectively) and positive ratio (region of positive ＜5%, 5-25%, 26-50%, 51-75% and ＞75% were assigned with scores of 0, 1, 2, 3 and 4, respectively).

Whole-cell or tissues were lysed with RIPA buffer (ThermoFisher, USA) supplemented with protease inhibitors and phosphatase inhibitors (ThermoFisher, USA) and quantified by BCA Protein Assay Kit (ThermoFisher, USA). Then the protein lysates were electrophoresed with 4-20% SDS-PAGE (GenScript, China) and transferred to 0.22 μm PVDF membranes (Merck, USA). Membranes were blocked with 5% BSA or nonfat milk for 2 h, and incubated with primary antibodies overnight. After washed with TBST and incubated with secondary antibodies, the protein bands were exposed with the chemiluminescence system (Biorad, USA). The antibodies were listed in Supplementary Table S6.

**Dual luciferase reporter assay**

Based on the prediction of SRAMP and RMBase version 2.0, partial sequences of *circSTX6* with potential m^6^A sites were cloned into the luciferase reporter vector as the wild-type plasmids (pcDNA3.1 vector with the firefly and renilla luciferase). Two adenosines (A) in the potential m^6^A sites were replaced by cytosines (C) as the mutant plasmids.

Similarly, based on the prediction of circBase, two potential internal ribosome entry sites (IRES) were cloned into the vectors as wild-type plasmids (IRES_239-342_ and IRES_245-391_), respectively.

Briefly, HCC cells were transfected in the 24-well plates with 0.6 μg wild-type or mutant plasmids. After 48 h, the luciferase activity was detected using Dual Luciferase Reporter Assay Kit (DL101-01, Vazyme), and the relative luciferase activity was based on “Firefly-luc/Renilla-luc”

Reporter sequences were listed as follows:

**hsa-circ-0007905 WT pmirGLO**

GACATGAAAGATCAGATGTCAACTTCATCTGTGCAGGCATTAGCTGAAAGAAAAAATAGACAGGCACTGCTGGGAGACAGTGGCAGCCAGAACTGGAGCACTGGAACAACAGATAAATATGGGCGTCTGGACCGAGAGCTCCAGAGAGCCAATTCTCATTTCATTGAGGAGCAGCAGGCACAGCAGCAGTTGATCGTGGAACAGCAGGATGAGCAGTTGGAGCTGGTCTCTGGCAGCATCGGGGTGCTGAAGAACATGTCCCAGCGCATCGGAGGGGAGCTGGAGGAACAGGCAGTTATGTTGGAAGATTTCTCTCACGAATTGGAGAGCACTCAGTCCCGGCTGGACAATGTGATGAAGAAACTTGCAAAAGTATCTCATATGACCAGTG

**hsa-circ-0007905 MUT pmirGLO**

GACATGAAAGATCAGATGTCAACTTCATCTGTGCAGGCATTAGCTGAAAGAAAAAATAGACAGGCACTGCTGGGAGACAGTGGCAGCCAGAACTGGAGCACTGGAACAACAGATAAATATGGGCGTCTGGACCGAGAGCTCCAGAGAGCCAATTCTCATTTCATTGAGGAGCAGCAGGCACAGCAGCAGTTGATCGTGGAACAGCAGGATGAGCAGTTGGAGCTGGTCTCTGGCAGCATCGGGGTGCTGAAGAACATGTCCCAGCGCATCGGAGGGGAGCTGGAGGAACAGGCAGTTATGTTGGAAGATTTCTCTCACGAATTGGAGAGCACTCAGTCCCGGCTGGCCAATGTGATGAAGAACCTTGCAAAAGTATCTCATATGACCAGTG

**IRES_239-342_**

TCGGGGTGCTGAAGAACATGTCCCAGCGCATCGGAGGGGAGCTGGAGGAACAGGCAGTTATGTTGGAAGATTTCTCTCACGAATTGGAGAGCACTCAGTCCCGG

**IRES2_245-391_**

TGCTGAAGAACATGTCCCAGCGCATCGGAGGGGAGCTGGAGGAACAGGCAGTTATGTTGGAAGATTTCTCTCACGAATTGGAGAGCACTCAGTCCCGGCTGGACAATGTGATGAAGAAACTTGCAAAAGTATCTCATATGACCAGTG

**Positive control**

GCCCCTCTCCCTCCCCCCCCCCTAACGTTACTGGCCGAAGCCGCTTGGAATAAGGCCGGTGTGCGTTTGTCTATATGTTATTTTCCACCATATTGCCGTCTTTTGGCAATGTGAGGGCCCGGAAACCTGGCCCTGTCTTCTTGACGAGCATTCCTAGGGGTCTTTCCCCTCTCGCCAAAGGAATGCAAGGTCTGTTGAATGTCGTGAAGGAAGCAGTTCCTCTGGAAGCTTCTTGAAGACAAACAACGTCTGTAGCGACCCTTTGCAGGCAGCGGAACCCCCCACCTGGCGACAGGTGCCTCTGCGGCCAAAAGCCACGTGTATAAGATACACCTGCAAAGGCGGCACAACCCCAGTGCCACGTTGTGAGTTGGATAGTTGTGGAAAGAGTCAAATGGCTCTCCTCAAGCGTATTCAACAAGGGGCTGAAGGATGCCCAGAAGGTACCCCATTGTATGGGATCTGATCTGGGGCCTCGGTACACATGCTTTACATGTGTTTAGTCGAGGTTAAAAAAACGTCTAGGCCCCCCGAACCACGGGGACGTGGTTTTCCTTTGAAAAACACGATGATAATATGGCCACAACC

**Negative control**

TTCATCTGTGCAGGCATTAGCTGAAAGAAAAAATAGACAGGCACTGCTGGGAGACAGTGGCAGCCAGAACTGGAGCACTGGAACAACAGATAAATATGGGCGTCTGGACCGAGAGCTCCAGAGAGCCAATTCTCATTTCATTGAGGAGCAGCAGGCACAGCAGCAGTTGATCGTGGAAC

**Cell proliferation assays**

Cell proliferation capacity was measured by Cell Counting Kit (CCK8, MCE) and colony formation assay. In terms of CCK8, 2.0×10^3^ HCC cells were seeded into 96-well plates with five duplicates. CCK8 reagents were diluted with DMEM at a ratio of 1:9 and 100 μL for each well. The 450 nm absorbance was detected after 1 h incubation at 37 ℃ in each day for consecutive five days, followed by the establishment of cell viability curves. In terms of colony formation, 2.0×10^3^ HCC cells were seeded into 6-well plates with three duplicates. After three weeks, cells were fixed with formalin and stained with crystal violet solution for observation.

**5-Ethynyl-20-deoxyuridine (EdU)**

EdU assay was employed with BeyoClick™ EdU Cell Proliferation Kit with Alexa Fluor 555 (Beyotime, China). A total of 1.0×10^5^ HCC cells were seeded into confocal plates and incubated with 50μM EdU solutions for 2 h at 37℃. Then they were fixed with formalin and permeabilized with 0.1% Triton X-100. Cells were subsequently incubated with Click reaction buffer and stained with Azide 555 and Hoechest 33342, followed by the visualization under fluorescence microscope.

**Apoptosis and cell cycle assays**

For apoptosis, HCC cells that transfected with si-*circSTX6* after 48 h were collected and washed by phosphate buffer saline (PBS), followed by the staining with Annexin V-FITC/PI Apoptosis Detection Kit (A211-01, Vazyme, China) for 15 min at 4℃. Then cell apoptosis of each group was evaluated by flow cytometry (BD, USA).

For cell cycle, HCC cells that transfected with si-*circSTX6* after 48 h were collected, washed and fixed with 70% ethanol at 4℃ overnight. The next day, cells were washed with cold PBS for several times and stained with PI (Vazyme, China) for 30 min at 4℃. Then cell cycle of each group was evaluated by flow cytometry (BD, USA) and the results were analyzed with FlowJo v10.8.1.

**Cell migration and invasion assays**

Transwell chambers (Falcon, USA) were precoated with (invasion) or without (migration) diluted matrigel (Corning, USA) and inserted into 24-well plates. A total of 5.0×10^4^ treated HCC cells were seeded into the upper chamber with 200 μL serum-free DMEM, while the 24-well plate were supplemented with 800 μL serum-sufficient DMEM in advance. Cells on the bottom layer of chambers were fixed and stained with Wright-Giemsa dye after 48 h for migration, and 72 h or longer for invasion.

**Wound healing migration assay**

5.0×10^4^ treated HCC cells were seeded into two wells of the two-chamber mould laid on 12-well plates, respectively. After 24 h, the mould was removed, the culture medium was changed into serum-free DMEM and the photograph of “area of gap (0h)” was taken. After 72 h, the photograph of “area of gap (72h)” was taken. The relative wound areas were measured by ImageJ software based on the formula of “area of gap (0h) – area of gap (72h)/ area of gap (0h).

**Subcutaneous tumor implantation and pulmonary metastasis experiments**

BALB/c nude mice (four-week old) were ordered from the Experimental Animal Center of Chinese Academy of Sciences (Shanghai, China). 5.0×10^6^ stably transfected HCC cells resuspended in 100 μL PBS were subcutaneously transplanted into the right flank of nude mice. The length and width of the tumors were measured every five days until 28 days later. Then the mice were sacrificed and the tumor weights were measured. The tumors were immediately fixed in formalin for further histology use. The growth curve of tumors was established based on the tumor volume calculated with the formula of “length×width^2^/2”.

2.0×10^6^ stably transfected HCC cells resuspended in 100 μL PBS were intravenously injected through the tail veins of mice. The mice were euthanized after 3 months or longer to harvest their lungs. The lung tissues were immediately fixed in formalin for further histology use.

All mice received humane care from experienced breeders according to the Guidelines for the Care and Use of Laboratory Animals. All the animal experiments were scrutinized and approved by the Ethics Committee for Laboratory Animals of the First Affiliated Hospital of Zhejiang University.

**RNA extraction and real time qPCR (RT-qPCR)**

Cell and tissue total RNA were extracted using RNA-Quick Purification Kit (Esunbio). Total RNA was reverse transcribed into cDNA with HiScript II Q Select RT SuperMix for qPCR (R233-01, Vazyme) and HiScript III All-in-one RT SuperMix Perfect for qPCR (R333-01, Vazyme). RT-qPCR was performed with ChamQ SYBR Green Master Mix (Vazyme) on QuantStudio 5 Real-Time PCR System (ThermoFisher). The relative cycle threshold (CT) value was computed with 2*^ΔΔCt^* and normalized to that of *GAPDH*. The primers of all genes were ordered from Tsingke Biological Technology and listed in Supplementary Table S5.

**RNA sequencing**

Total RNAs were extracted from the MHCC97H cells that transfected with si-*circSTX6*#2 and negative control (NC), respectively. Triplicate samples were assigned in each group and the knockdown efficiency was prechecked by qPCR. The RNA-sequencing was conducted by LC-Bio (Hangzhou, China) based on their Illumina sequence platform (Illumina). P < 0.05 was set as the criteria for differentially expressed genes (DEG) and log_2_ (fold change) >1 or <-1. Kyoto Encyclopedia of Genes and Genomes (KEGG) enrichment analysis were conducted on DEGs.

**RNA pulldown, silver staining and mass spectrum (MS)**

RNA pulldown was conducted with the Pierce Magnetic RNA-Protein Pull-Down Kit (#20164, ThermoFisher) in accordance with the protocols. Streptavidin magnetic beads were precoated with the biotinylated *circSTX6* probes (RepoBio, Hangzhou, China) targeting *circSTX6* junction sites and *cricSTX6* segment probes. Then the MHCC97H and HCCLM3 cell lysates were incubated with the beads at 4 ℃ overnight. The next day, the RBPs were eluted after several washes and used for western blot. The sequences of *circSTX6* segment probes and *circSTX6* probes and NC probe were listed below.

After RNA pulldown, the target proteins were electrophoresed with 4-20% SDS-PAGE and the gel was used for silver staining with Fast Silver Stain Kit (Beyotime, China). Based on the variance between NC-probe group and *circSTX6*-probe group, target bands were excised with clean scalpel blade and sent for MS analysis (OE Bio, China). Proteins identification and quantification was analyzed with Proteome Discoverer software (ThermoFisher). The silver staining and MS analysis of *circSTX6* OE followed the same procedures.

**RNA pulldown probes**

The probe sequences for circSTX6 were listed as follows:

*CircSTX6*: Biotin-5’- ATCTTTCATGTCCACTGGTCAT-3’;

*CircSTX6* segment 1: Biotin-5’-TCTGATCTTTCATGTCCACTGGTCA-3’;

*CircSTX6* segment 2: Biotin-5’-GTGCCTGCTGCTCCTCAATGAAAT-3’;

*CircSTX6* segment 3: Biotin-5’-TCATGTCCACTGGTCATATGAGAT-3’;

Negative control probe: Biotin-5’-GTGTAACACGTCTATACGCCCA-3’.

**Fluorescence in situ hybridization (FISH) and immunofluorescence (IF)**

The RNA-FISH assay was performed with the Fluorescence in Situ Hybridization Kit by RiboBio (C10910) and fully followed the kit protocols. The fluorescently labeled *circSTX6* FISH probe was designed and synthesized by RepoBio (Hangzhou, China). The cells were seeded into the confocal plates at a density of 6×10^4^ cells per well. Then they were fixed with 4% paraformaldehyde, permeabilized with 1% Triton X-100 and incubated with Cy3-labeled *circSTX6* probe overnight in 37℃. The next day, after several washes, the cells were stained with DAPI. For FISH/IF assay, after incubation with the *circSTX6* probe, cells were also incubated with HNRNPD antibodies (ABclonal) overnight at 4℃. The next day, after several washes with PBS, cells were incubated with secondary antibodies (green fluorescent) for 30 min at 37℃ and stained with DAPI. The images were acquired by the confocal microscopy (Leica LAS X, Germany). The probe sequences for *circSTX6* were listed as follows: hsa_circ_0007905-FISH-1: Cy3-5’-TCTGATCTTTCATGTCCACTGGTCATATGA-3’; hsa_circ_0007905-FISH-2: Cy3-5’-ATCTTTCATGTCCACTGGTCAT-3’.

**RNA immunoprecipitation (RIP) and HNRNPD truncation**

The RIP assay was conducted with PureBinding^®^ RNA Immunoprecipitation Kit (P0101, GeneSeed, China) following the manufacturer’s manual. Briefly, the antibodies (5 μg) targeting HNRNPD, Flag and corresponding IgG were pre-captured by magnetic beads. Then the prepared protein lysates were incubated with the beads at 4℃ for overnight and treated with proteinase K. The next day, target RNAs were eluted and purified for qPCR. Relative enrichment was calculated and normalized based on the input.

In order to explore the binding motifs of HNRNPD with *circSTX6*, truncation plasmids were constructed based on the RRM1 and RRM2 domains of HNRNPD by RepoBio (Hangzhou, China). All plasmid sequences were labeled with Flag tag. The transfection efficiency and truncation were checked by Western blot to match their predicted molecular weight. The truncation sequences were listed:

**HNRNPD-WT** **pcDNA3.1(+)**

GCTAGCGCCGCCACCATGTCGGAGGAGCAGTTCGGCGGGGACGGGGCGGCGGCAGCGGCAACGGCGGCGGTAGGCGGCTCGGCGGGCGAGCAGGAGGGAGCCATGGTGGCGGCGACACAGGGGGCAGCGGCGGCGGCGGGAAGCGGAGCCGGGACCGGGGGCGGAACCGCGTCTGGAGGCACCGAAGGGGGCAGCGCCGAGTCGGAGGGGGCGAAGATTGACGCCAGTAAGAACGAGGAGGATGAAGGCCATTCAAACTCCTCCCCACGACACTCTGAAGCAGCGACGGCACAGCGGGAAGAATGGAAAATGTTTATAGGAGGCCTTAGCTGGGACACTACAAAGAAAGATCTGAAGGACTACTTTTCCAAATTTGGTGAAGTTGTAGACTGCACTCTGAAGTTAGATCCTATCACAGGGCGATCAAGGGGTTTTGGCTTTGTGCTATTTAAAGAATCGGAGAGTGTAGATAAGGTCATGGATCAAAAAGAACATAAATTGAATGGGAAGGTGATTGATCCTAAAAGGGCCAAAGCCATGAAAACAAAAGAGCCGGTTAAAAAAATTTTTGTTGGTGGCCTTTCTCCAGATACACCTGAAGAGAAAATAAGGGAGTACTTTGGTGGTTTTGGTGAGGTGGAATCCATAGAGCTCCCCATGGACAACAAGACCAATAAGAGGCGTGGGTTCTGCTTTATTACCTTTAAGGAAGAAGAACCAGTGAAGAAGATAATGGAAAAGAAATACCACAATGTTGGTCTTAGTAAATGTGAAATAAAAGTAGCCATGTCGAAGGAACAATATCAGCAACAGCAACAGTGGGGATCTAGAGGAGGATTTGCAGGAAGAGCTCGTGGAAGAGGTGGTGGCCCCAGTCAAAACTGGAACCAGGGATATAGTAACTATTGGAATCAAGGCTATGGCAACTATGGATATAACAGCCAAGGTTACGGTGGTTATGGAGGATATGACTACACTGGTTACAACAACTACTATGGATATGGTGATTATAGCAACCAGCAGAGTGGTTATGGGAAGGTATCCAGGCGAGGTGGTCATCAAAATAGCTACAAACCATACGATTACAAGGACGACGATGACAAGTAGCTCGAG

**HNRNPD-del RPM1 pcDNA3.1(+)：**

GCTAGCGCCGCCACCATGTCGGAGGAGCAGTTCGGCGGGGACGGGGCGGCGGCAGCGGCAACGGCGGCGGTAGGCGGCTCGGCGGGCGAGCAGGAGGGAGCCATGGTGGCGGCGACACAGGGGGCAGCGGCGGCGGCGGGAAGCGGAGCCGGGACCGGGGGCGGAACCGCGTCTGGAGGCACCGAAGGGGGCAGCGCCGAGTCGGAGGGGGCGAAGATTGACGCCAGTAAGAACGAGGAGGATGAAGGCCATTCAAACTCCTCCCCACGACACTCTGAAGCAGCGACGGCACAGCGGGAAGAACCGGTTAAAAAAATTTTTGTTGGTGGCCTTTCTCCAGATACACCTGAAGAGAAAATAAGGGAGTACTTTGGTGGTTTTGGTGAGGTGGAATCCATAGAGCTCCCCATGGACAACAAGACCAATAAGAGGCGTGGGTTCTGCTTTATTACCTTTAAGGAAGAAGAACCAGTGAAGAAGATAATGGAAAAGAAATACCACAATGTTGGTCTTAGTAAATGTGAAATAAAAGTAGCCATGTCGAAGGAACAATATCAGCAACAGCAACAGTGGGGATCTAGAGGAGGATTTGCAGGAAGAGCTCGTGGAAGAGGTGGTGGCCCCAGTCAAAACTGGAACCAGGGATATAGTAACTATTGGAATCAAGGCTATGGCAACTATGGATATAACAGCCAAGGTTACGGTGGTTATGGAGGATATGACTACACTGGTTACAACAACTACTATGGATATGGTGATTATAGCAACCAGCAGAGTGGTTATGGGAAGGTATCCAGGCGAGGTGGTCATCAAAATAGCTACAAACCATACGATTACAAGGACGACGATGACAAGTAGCTCGAG

**HNRNPD-del RPM2 pcDNA3.1(+)：**

GCTAGCGCCGCCACCATGTCGGAGGAGCAGTTCGGCGGGGACGGGGCGGCGGCAGCGGCAACGGCGGCGGTAGGCGGCTCGGCGGGCGAGCAGGAGGGAGCCATGGTGGCGGCGACACAGGGGGCAGCGGCGGCGGCGGGAAGCGGAGCCGGGACCGGGGGCGGAACCGCGTCTGGAGGCACCGAAGGGGGCAGCGCCGAGTCGGAGGGGGCGAAGATTGACGCCAGTAAGAACGAGGAGGATGAAGGCCATTCAAACTCCTCCCCACGACACTCTGAAGCAGCGACGGCACAGCGGGAAGAATGGAAAATGTTTATAGGAGGCCTTAGCTGGGACACTACAAAGAAAGATCTGAAGGACTACTTTTCCAAATTTGGTGAAGTTGTAGACTGCACTCTGAAGTTAGATCCTATCACAGGGCGATCAAGGGGTTTTGGCTTTGTGCTATTTAAAGAATCGGAGAGTGTAGATAAGGTCATGGATCAAAAAGAACATAAATTGAATGGGAAGGTGATTGATCCTAAAAGGGCCAAAGCCATGAAAACAAAAGAGCCGGTTCAATATCAGCAACAGCAACAGTGGGGATCTAGAGGAGGATTTGCAGGAAGAGCTCGTGGAAGAGGTGGTGGCCCCAGTCAAAACTGGAACCAGGGATATAGTAACTATTGGAATCAAGGCTATGGCAACTATGGATATAACAGCCAAGGTTACGGTGGTTATGGAGGATATGACTACACTGGTTACAACAACTACTATGGATATGGTGATTATAGCAACCAGCAGAGTGGTTATGGGAAGGTATCCAGGCGAGGTGGTCATCAAAATAGCTACAAACCATACGATTACAAGGACGACGATGACAAGTAGCTCGAG

**HNRNPD-del RPM1+2 pcDNA3.1(+)：**

GCTAGCGCCGCCACCATGTCGGAGGAGCAGTTCGGCGGGGACGGGGCGGCGGCAGCGGCAACGGCGGCGGTAGGCGGCTCGGCGGGCGAGCAGGAGGGAGCCATGGTGGCGGCGACACAGGGGGCAGCGGCGGCGGCGGGAAGCGGAGCCGGGACCGGGGGCGGAACCGCGTCTGGAGGCACCGAAGGGGGCAGCGCCGAGTCGGAGGGGGCGAAGATTGACGCCAGTAAGAACGAGGAGGATGAAGGCCATTCAAACTCCTCCCCACGACACTCTGAAGCAGCGACGGCACAGCGGGAAGAACCGGTTCAATATCAGCAACAGCAACAGTGGGGATCTAGAGGAGGATTTGCAGGAAGAGCTCGTGGAAGAGGTGGTGGCCCCAGTCAAAACTGGAACCAGGGATATAGTAACTATTGGAATCAAGGCTATGGCAACTATGGATATAACAGCCAAGGTTACGGTGGTTATGGAGGATATGACTACACTGGTTACAACAACTACTATGGATATGGTGATTATAGCAACCAGCAGAGTGGTTATGGGAAGGTATCCAGGCGAGGTGGTCATCAAAATAGCTACAAACCATACGATTACAAGGACGACGATGACAAGTAGCTCGAG

**Sequences of *circSTX6* with ATG mutation (*circSTX6*-MUT) pcDNA3.1 and linear circSTX6-144aa pcDNA3.1 overexpression plasmids**

**hsa-circ-0007905 mut pcDNA3.1(+):**

GAATTCAAAGTGCTGAGATTACAGGCGTGAGCCACCACCCCCGGCCCACTTTTTGTAAAGGTACGTACTAATGACTTTTTTTTTATACTTCAGGACATGGATTACAAGGACGACGATGACAAGTGAAAAGATCAGATGTCAACTTCATCTGTGCAGGCATTAGCTGAAAGAAAAAATAGACAGGCACTGCTGGGAGACAGTGGCAGCCAGAACTGGAGCACTGGAACAACAGATAAATATGGGCGTCTGGACCGAGAGCTCCAGAGAGCCAATTCTCATTTCATTGAGGAGCAGCAGGCACAGCAGCAGTTGATCGTGGAACAGCAGGATGAGCAGTTGGAGCTGGTCTCTGGCAGCATCGGGGTGCTGAAGAACATGTCCCAGCGCATCGGAGGGGAGCTGGAGGAACAGGCAGTTATGTTGGAAGATTTCTCTCACGAATTGGAGAGCACTCAGTCCCGGCTGGACAATGTGATGAAGAAACTTGCAAAAGTATCTCATATGACCAGTGGTAAGAAGCAAGGAAAAGAATTAGGCTCGGCACGGTAGCTCACACCTGTAATCCCAGCAGCGGCCGC

**Linear circSTX6-144aa pcDNA3.1(+):**

ATGAAAGATCAGATGTCAACTTCATCTGTGCAGGCATTAGCTGAAAGAAAAAATAGACAGGCACTGCTGGGAGACAGTGGCAGCCAGAACTGGAGCACTGGAACAACAGATAAATATGGGCGTCTGGACCGAGAGCTCCAGAGAGCCAATTCTCATTTCATTGAGGAGCAGCAGGCACAGCAGCAGTTGATCGTGGAACAGCAGGATGAGCAGTTGGAGCTGGTCTCTGGCAGCATCGGGGTGCTGAAGAACATGTCCCAGCGCATCGGAGGGGAGCTGGAGGAACAGGCAGTTATGTTGGAAGATTTCTCTCACGAATTGGAGAGCACTCAGTCCCGGCTGGACAATGTGATGAAGAAACTTGCAAAAGTATCTCATATGACCAGTGGACATGAAAGATCAGATGTCAACTTCATCTGTGCAGGCATTAGCTGA

**Supplementary Figures**


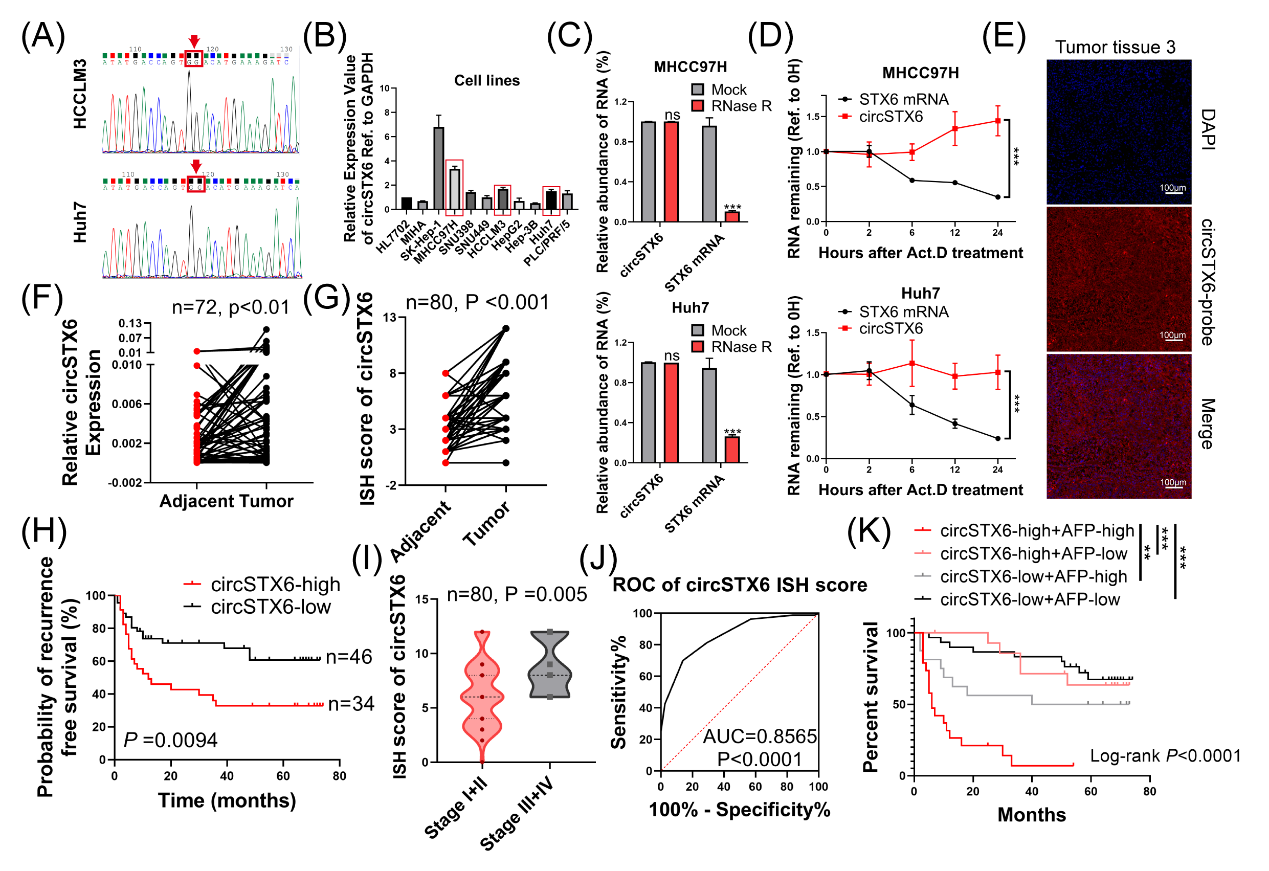


**Supplementary Figure 1.** Characterization and clinical relevance of *circSTX6*. (A) Sanger sequencing detected spliced junction sites in HCCLM3 and Huh7 cells. (B) The expression of *circSTX6* were detected in two normal hepatocytes and nine HCC cell lines by qPCR. (C) Expression of *circSTX6* and *STX6* mRNA were detected by qPCR following RNase R treatment (3U/ug). (D) Expression of *circSTX6* and *STX6* mRNA were detected by qPCR at indicated time points following actinomycin D treatment (10 μg/mL). (E) FISH assay showing the subcellular distribution of *circSTX6* in HCC tumor tissues. (F) Relative *circSTX6* expression detected by qPCR were examined by paired *t* test. (G) ISH score of 80 pairs of tumor and adjacent normal tissues were shown. (H) Kaplan-Meier prognostic analyses showing the RFS (n = 80, *P* = 0.0094) of HCC patients based on *circSTX6* expression. (I) ISH score in different stages of HCC patients were compared. (J) Diagnostic ROC curve based on the ISH score of *circSTX6* in HCC corhort-3 (AUC = 0.8565, *P* < 0.0001). (K) Overall survival analysis based on the co-expression of *circSTX6* and AFP in HCC cohor-3 (n = 80, *P* < 0.0001).


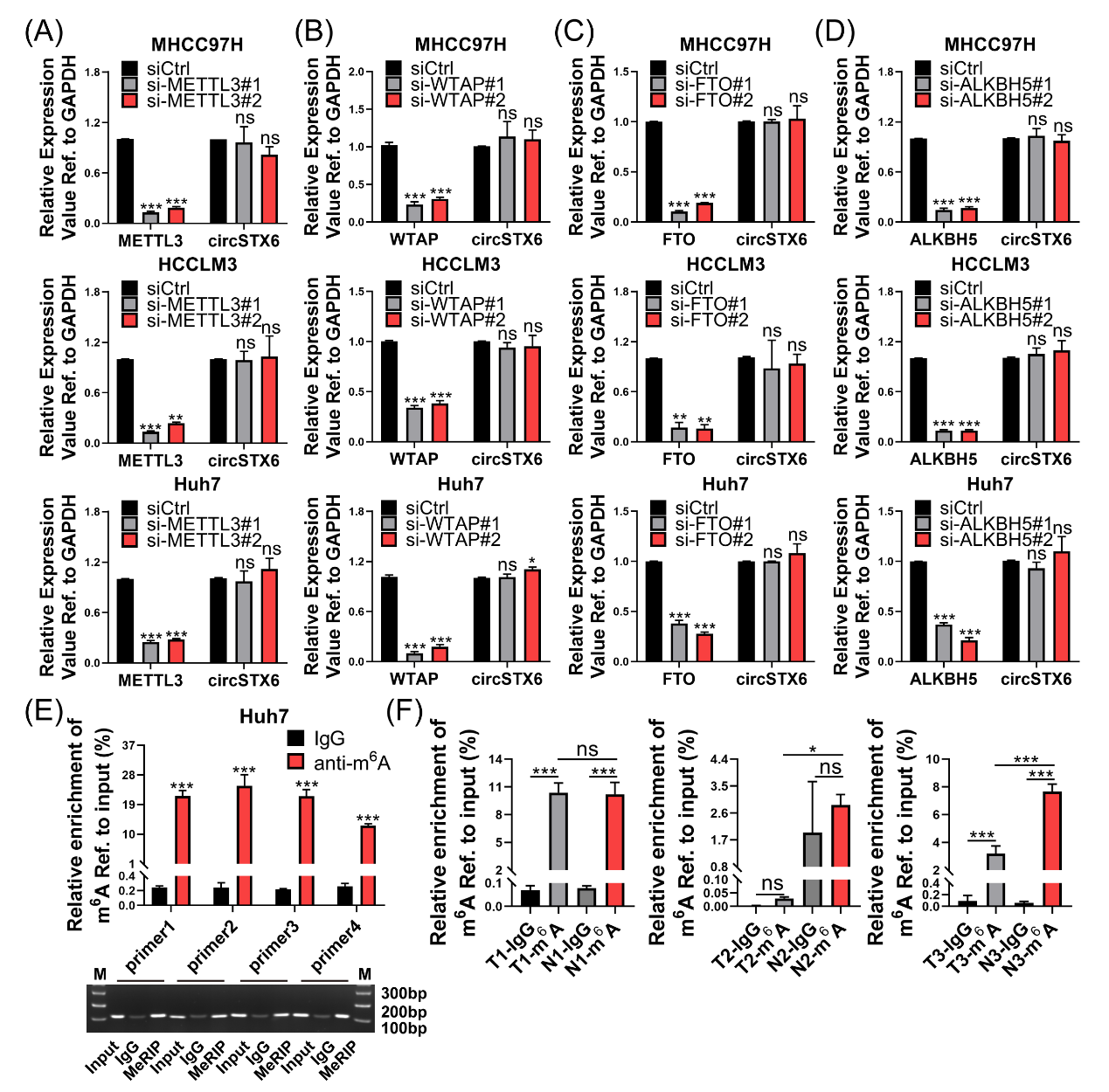


**Supplementary Figure 2.** The expression of *circSTX6* is regulated by METTL14. (A) The expression of *circSTX6* upon METTL3 knockdown in HCC cells. (B) The expression of *circSTX6* upon WTAP knockdown in HCC cells. (C) The expression of *circSTX6* upon FTO knockdown in HCC cells. (D) The expression of *circSTX6* upon ALKBH5 knockdown in HCC cells. (E) MeRIP assays were conducted in Huh7 cells and measured by qPCR. Relative enrichment by m^6^A-antibody was normalized based on input. Results of agarose gel electrophoresis using PCR products were shown. (F) MeRIP assays were conducted in three pairs of HCC tumor and adjacent normal tissues and the results measured by qPCR.


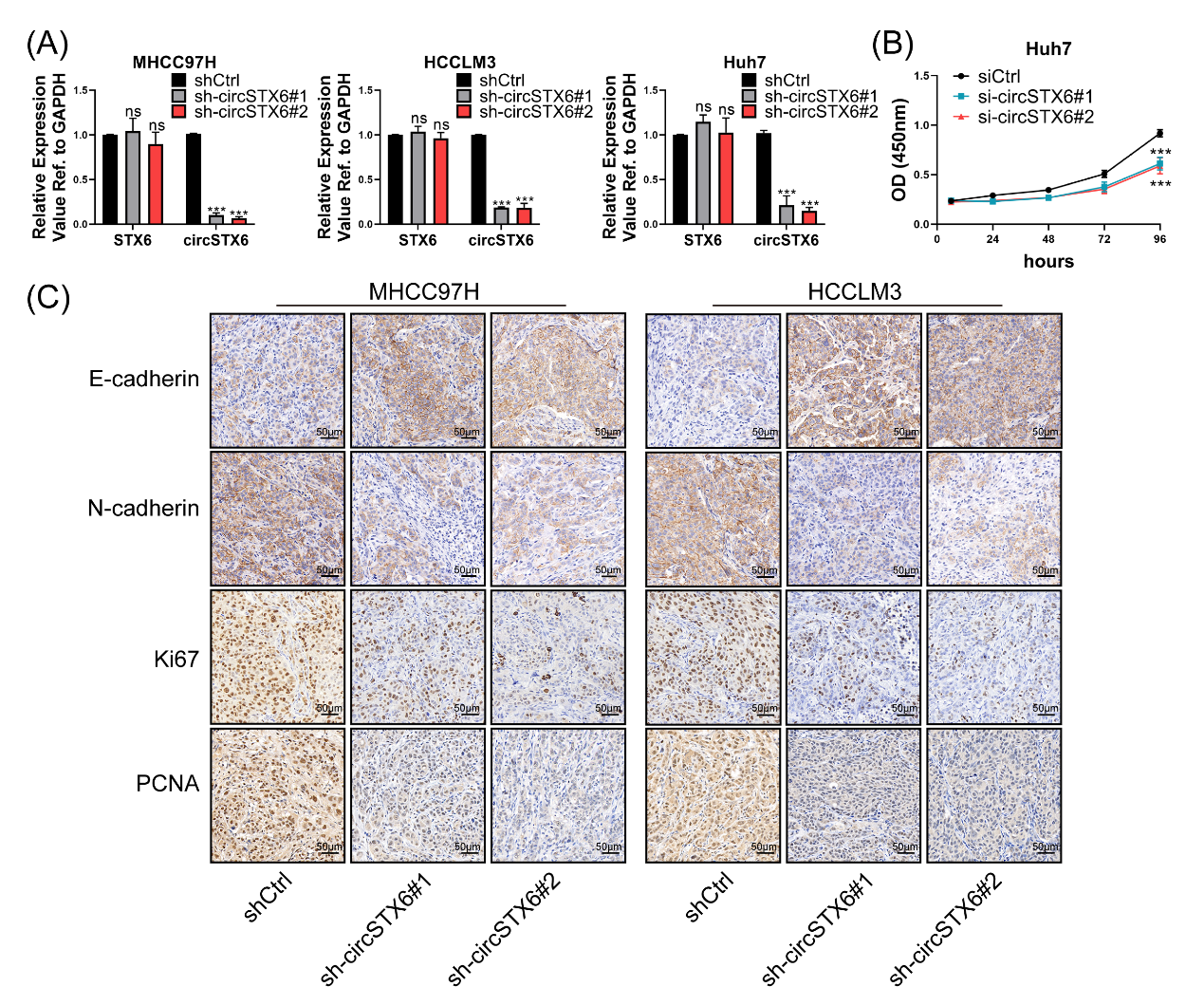


**Supplementary Figure 3.** Silencing of *circSTX6* inhibits HCC proliferation. (A) The knockdown efficiency of *circSTX6* in HCC cells transfected with *circSTX6* shRNAs. (B) CCK-8 assay showing the growth curve of Huh7 cells upon *circSTX6* knockdown. (C) Representative images of IHC staining in subcutaneous tumors. The IHC indicators included E-cadherin, N-cadherin, Ki67 and PCNA. Scale bar, 50 μm.


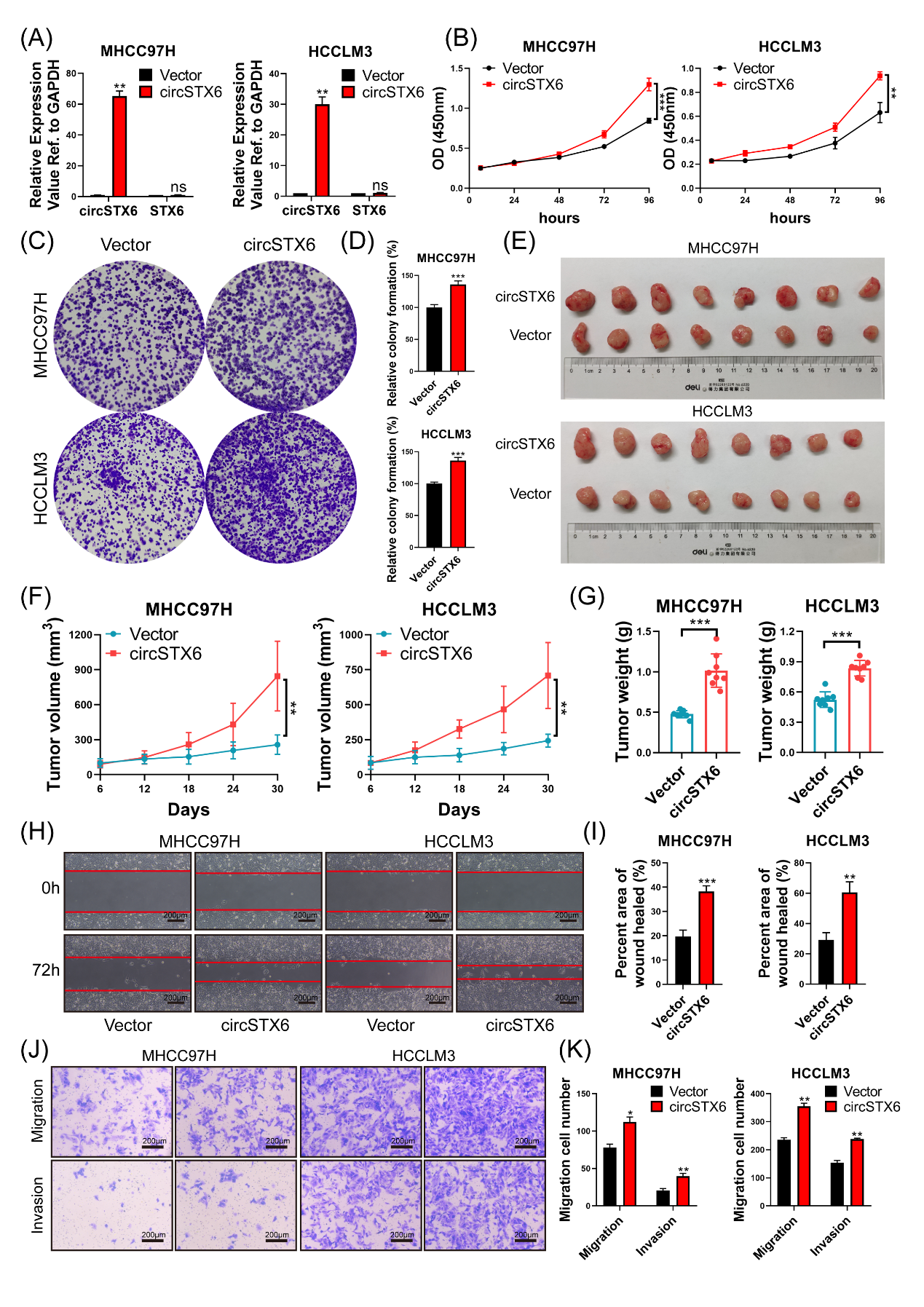


**Supplementary Figure 4.** Overexpression of *circSTX6* promotes HCC proliferation, migration and invasion *in vitro* and *in vivo*. (A) The efficiency of *circSTX6* overexpression was validated by qPCR in MHCC97H and HCCLM3 cells. (B) The proliferation of HCC cells upon *circSTX6* overexpression was detected by CCK-8 assays. (C) The proliferation of HCC cells upon *circSTX6* overexpression was detected by colony formation assays. (D) Diagram showing quantified relative colony numbers.(E-G) Representative images of subcutaneous tumors in *circSTX6*-overexpression and control groups (n=8), and the statistical analysis of growth curves and tumor weight. (H and I) Wound healing assays showing migration capabilities of MHCC97H and HCCLM3 cells upon *circSTX6* overexpression. The wound-healed area was quantified in histograms. Scale bar, 200 μm. (J and K) The migration and invasion of HCC cells were detected by transwell assays upon *circSTX6* overexpression. The count of migrated cells was quantified in histograms. Scale bar, 200 μm.


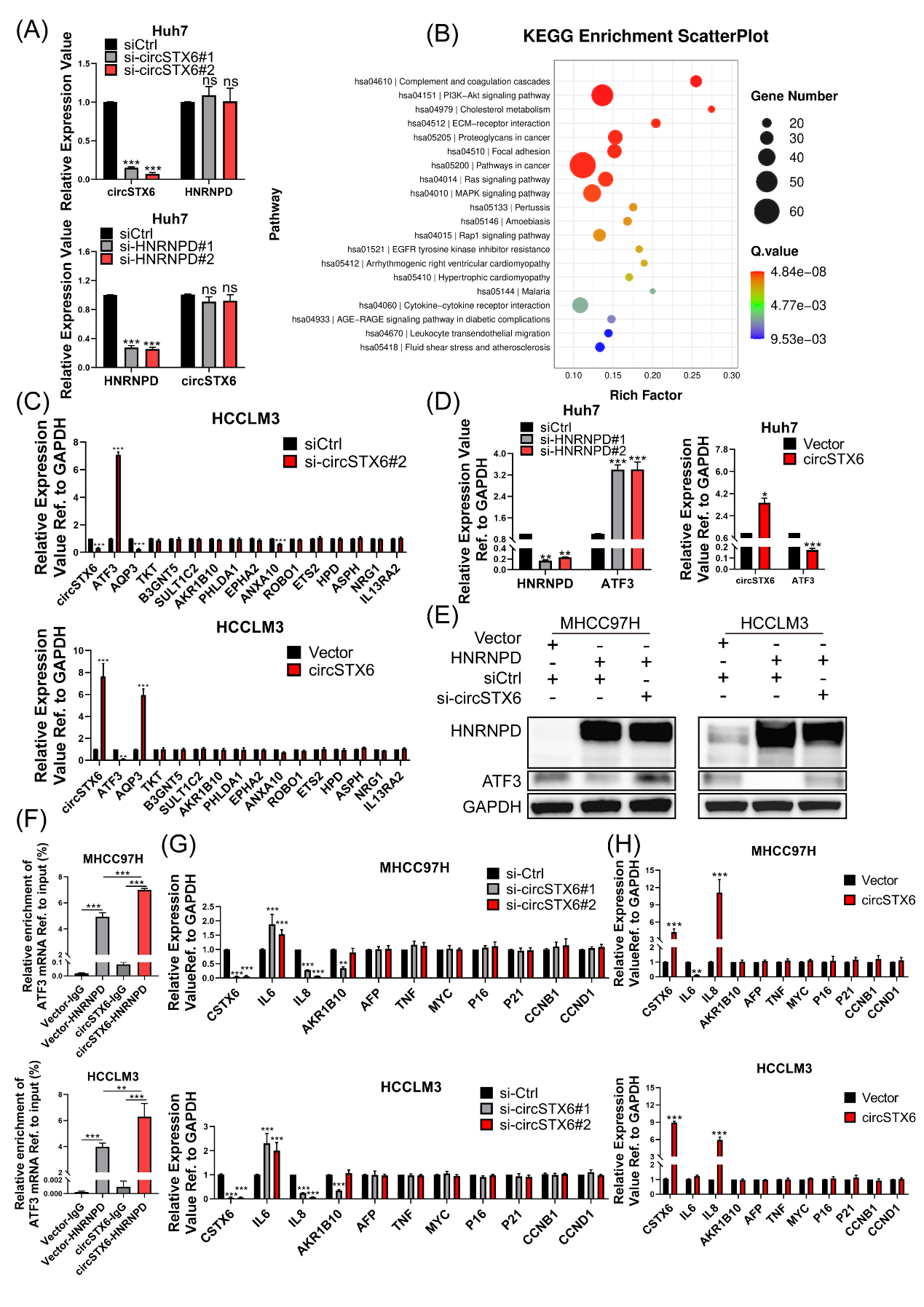


**Supplementary Figure 5.** Identification of downstream targets of *circSTX6*. (A) The expression of HNRNPD upon *circSTX6* knockdown and *circSTX6* upon HNRNPD knockdown were determined by qPCR in Huh7 cells. (B) KEGG enrichment analysis showed the enrichment of pathways including Pathways in cancer, PI3K-AKT signaling pathway and MAPK signaling pathway. (C) The expression of 15 candidate genes were preliminarily examined by qPCR in HCCLM3 cells. (D) The expression of *ATF3* was determined by qPCR upon HNRNPD knockdown or *circSTX6* overexpression. (E) The expression of ATF3 was examined by overexpression of HNRNPD in the presence or absence of *circSTX6* with Western blot. (F) HNRNPD-RIP assay was performed in *circSTX6*-overexpression or control HCC cells, followed by qPCR to detect the enrichment of *ATF3* mRNA. (G) The expression of 10 downstream targets of HNRNPD upon *circSTX6* silencing were examined by qPCR in HCC cells. (H) The expression of 10 downstream targets of HNRNPD upon *circSTX6* overexpression were examined by qPCR in HCC cells.


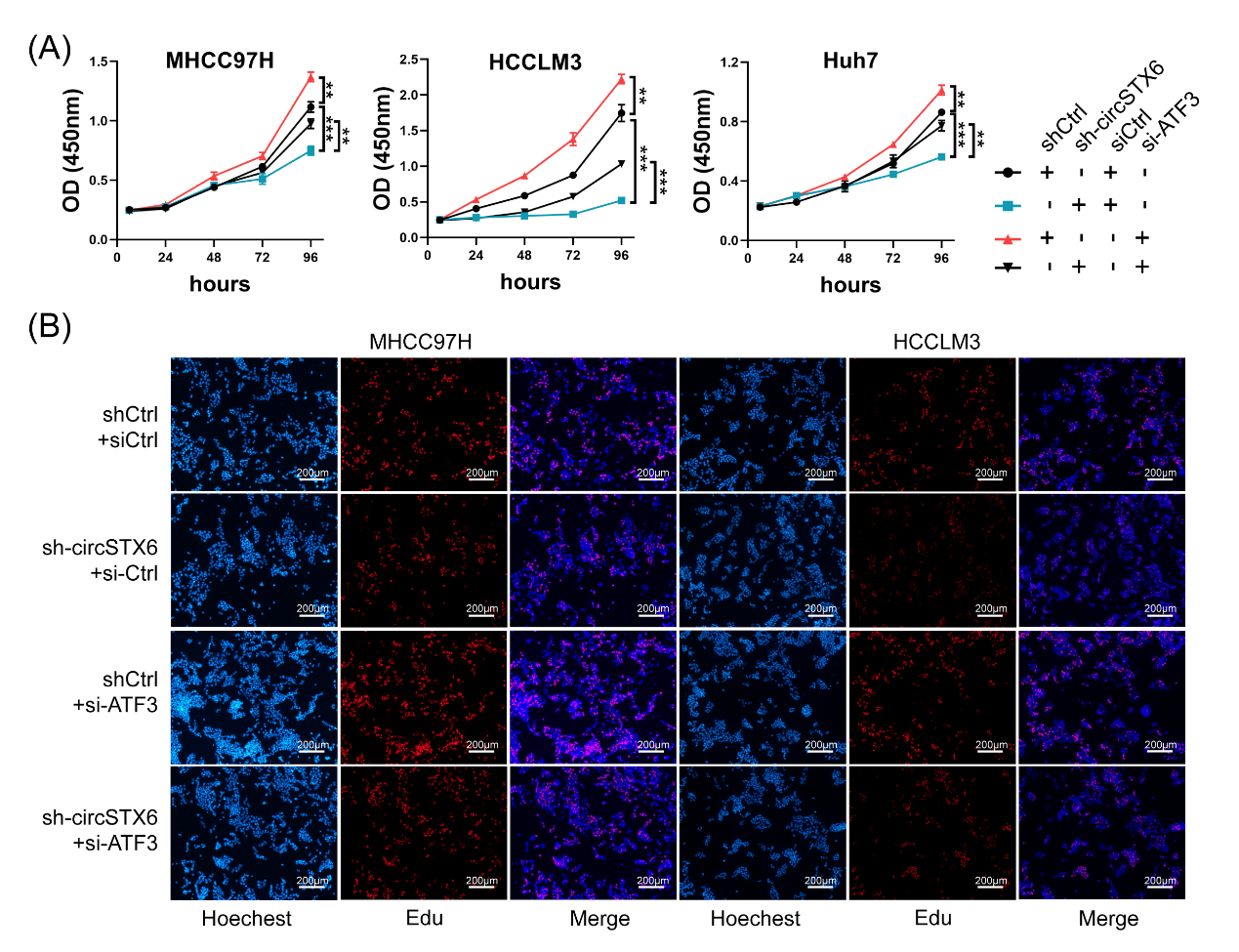


**Supplementary Figure 6.** Inhibition of ATF3 retrieves impaired proliferation of HCC cells with *circSTX6* deficiency. (A) CCK-8 assay was conducted to assess the proliferation of MHCC97H, HCCLM3 and Huh7 cells with either ATF3-inhibition or *circSTX6*-kncodown. (B) Representative images of EdU assays in either ATF3-silencing or *circSTX6*-dificiency HCC cells. Scale bar, 200 μm.


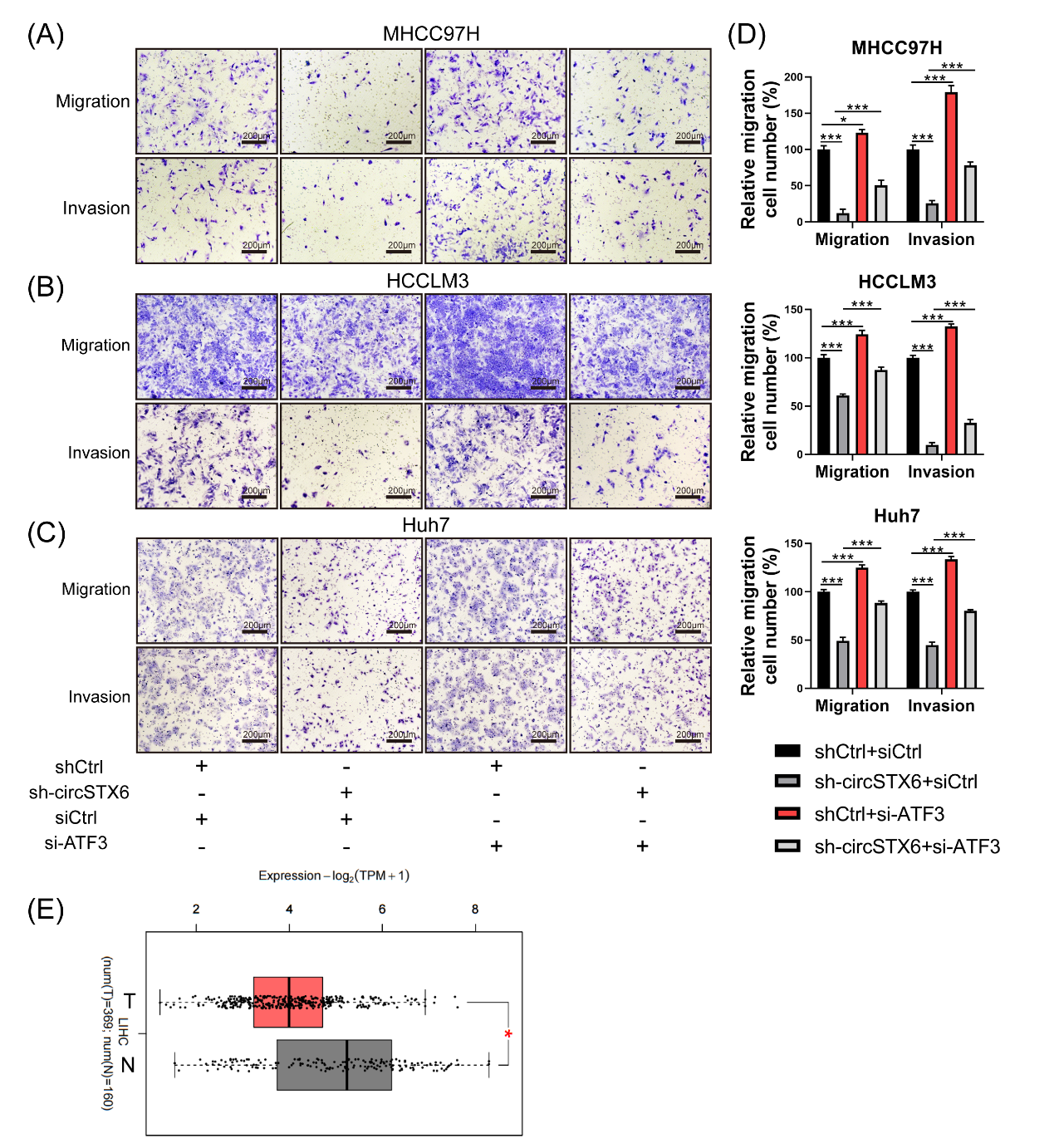


**Supplementary Figure 7.** Suppression of ATF3 restored impaired migration and invasion of HCC cells caused by *circSTX6* knockdown. (A-C) Migration and invasion capacities in MHCC97H, HCCLM3 and Huh7 cells with either ATF3-silencing or *circSTX6*-dificiency were evaluated by transwell assays. Scale bar, 200 μm. (D) Relative migration numbers of HCC cells in transwell assays were quantified in histograms. (E) Expression of *ATF3* in tumor and adjacent tissues based on TCGA HCC cohort (n=369) was analyzed by GEPIA software.


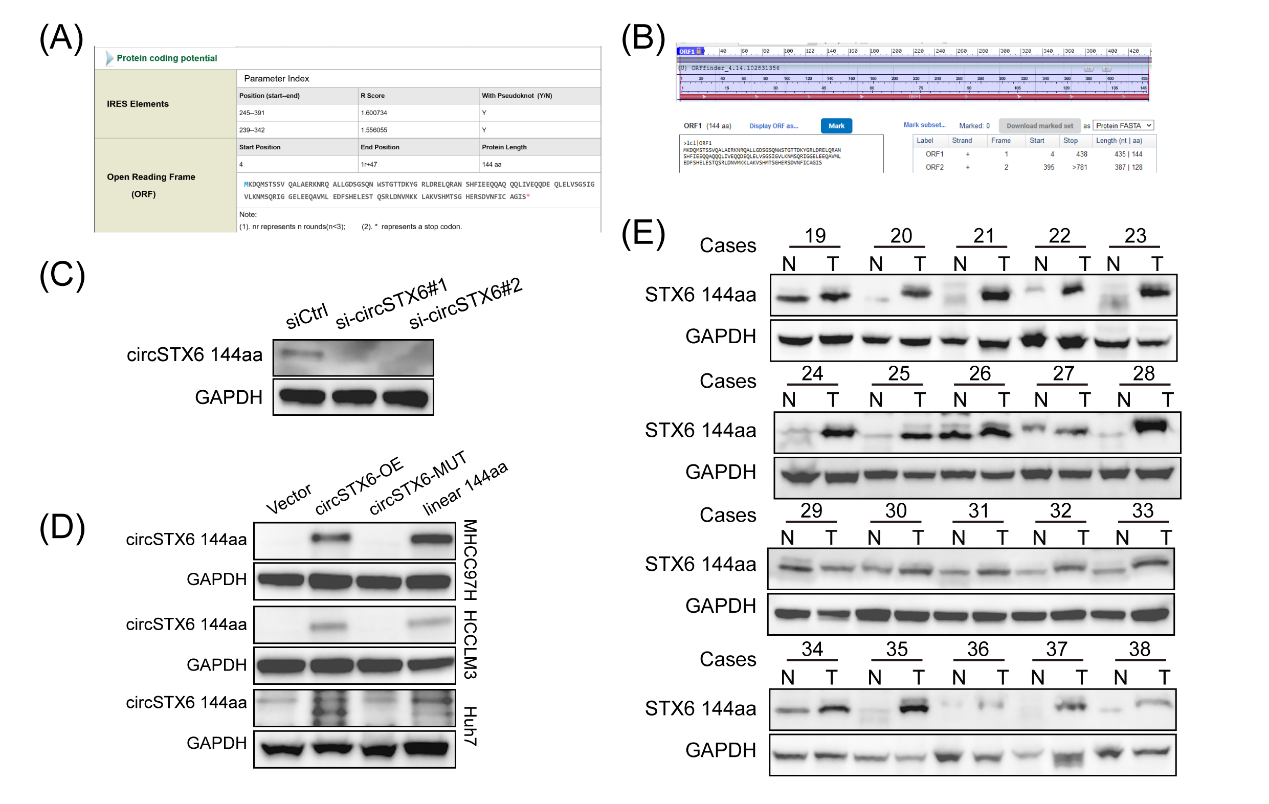


**Supplementary Figure 8.** *CircSTX6* encodes circSTX6-144aa. (A) Prediction of the potential IRES and ORF sequences on *circSTX6* by circRNADb software. (B) Prediction of potential ORF sequences based on *circSTX6* sequences by ORF finder software. (C) The expression of circSTX6-144aa in circSTX6-knockdown or negative control Huh7 cells was detected by Western blotting. (D) The expression of circSTX6-144aa was detected in HCC cells transfected with vector, *circSTX6*, *circSTX6* with ATG mutant, and linear circSTX6-144aa plasmids. (E) Expression of circSTX6-144aa was detected in 20 pairs of HCC tumor and adjacent normal tissues.

**Supplementary tables**

| \| **Table S1.** Clinicopathological characteristics of 72 HCC patients (HCC cohort-1) based on *circSTX6* expression. \| \| \| \| \| \| \| --- \| --- \| --- \| --- \| --- \| --- \| \| Characteristics \| *circSTX6*-high \| *circSTX6*-  low \| n \| *X²* \| *P* \| \| All cases \| 36 \| 36 \|  \|  \|  \| \| Gender \|  \|  \|  \| 0.7579 \| 0.3840 \| \| Male \| 30 \| 27 \| 57 \|  \|  \| \| Female \| 6 \| 9 \| 15 \|  \|  \| \| Age(years) \|  \|  \|  \| 0.5515 \| 0.4577 \| \| >60 \| 14 \| 11 \| 25 \|  \|  \| \| ≤60 \| 22 \| 25 \| 47 \|  \|  \| \| Tumor differentiation \|  \|  \|  \| 5.143 \| 0.0233* \| \| G1+G2 \| 24 \| 32 \| 56 \|  \|  \| \| G3 \| 12 \| 4 \| 16 \|  \|  \| \| Stage \|  \|  \|  \| 2.683 \| 0.1015 \| \| Ⅰ+Ⅱ \| 28 \| 33 \| 61 \|  \|  \| \| Ⅲ+Ⅳ \| 8 \| 3 \| 11 \|  \|  \| \| Tumor size \|  \|  \|  \| 4.677 \| 0.0306* \| \| >5 \| 26 \| 17 \| 43 \|  \|  \| \| ≤5 \| 10 \| 19 \| 29 \|  \|  \|  \| **Table S2.** Clinicopathological characteristics of 40 HCC patients (HCC cohort-2) based on *circSTX6* expression. \| \| \| \| \| \| \| --- \| --- \| --- \| --- \| --- \| --- \| \| Characteristics \| *circSTX6*-high \| *circSTX6*-  low \| n \| *X²* \| *P* \| \| All cases \| 27 \| 13 \|  \|  \|  \| \| Gender \|  \|  \|  \| 0.4070 \| 0.5235 \| \| Male \| 23 \| 12 \| 35 \|  \|  \| \| Female \| 4 \| 1 \| 5 \|  \|  \| \| Age(years) \|  \|  \|  \| 0.6089 \| 0.4352 \| \| >60 \| 11 \| 7 \| 18 \|  \|  \| \| ≤60 \| 16 \| 6 \| 22 \|  \|  \| \| Tumor differentiation \|  \|  \|  \| 3.077 \| 0.0794 \| \| G1+G2 \| 18 \| 12 \| 30 \|  \|  \| \| G3 \| 9 \| 1 \| 10 \|  \|  \| \| Stage \|  \|  \|  \| 0.1140 \| 0.7357 \| \| Ⅰ+Ⅱ \| 24 \| 12 \| 36 \|  \|  \| \| Ⅲ+Ⅳ \| 3 \| 1 \| 4 \|  \|  \| \| Tumor size \|  \|  \|  \| 0.01899 \| 0.8904 \| \| >5 \| 11 \| 5 \| 16 \|  \|  \| \| ≤5 \| 16 \| 8 \| 24 \|  \|  \|   **Table S3.** Clinicopathological characteristics of 80 HCC patients (HCC cohort-3) based on *circSTX6* expression. | | | | | |
| --- | --- | --- | --- | --- | --- | --- | --- | --- | --- | --- | --- | --- | --- | --- | --- | --- | --- | --- | --- | --- | --- | --- | --- | --- | --- | --- | --- | --- | --- | --- | --- | --- | --- | --- | --- | --- | --- | --- | --- | --- | --- | --- | --- | --- | --- | --- | --- | --- | --- | --- | --- | --- | --- | --- | --- | --- | --- | --- | --- | --- | --- | --- | --- | --- | --- | --- | --- | --- | --- | --- | --- | --- | --- | --- | --- | --- | --- | --- | --- | --- | --- | --- | --- | --- | --- | --- | --- | --- | --- | --- | --- | --- | --- | --- | --- | --- | --- | --- | --- | --- | --- | --- | --- | --- | --- | --- | --- | --- | --- | --- | --- | --- | --- | --- | --- | --- | --- | --- | --- | --- | --- | --- | --- | --- | --- | --- | --- | --- | --- | --- | --- | --- | --- | --- | --- | --- | --- | --- | --- | --- | --- | --- | --- | --- | --- | --- | --- | --- | --- | --- | --- | --- | --- | --- | --- | --- | --- | --- | --- | --- | --- | --- | --- | --- | --- | --- | --- | --- | --- | --- | --- | --- | --- | --- | --- | --- | --- | --- | --- | --- | --- | --- | --- | --- | --- | --- | --- | --- | --- | --- | --- | --- | --- | --- | --- | --- | --- | --- | --- | --- | --- | --- | --- | --- | --- | --- | --- | --- | --- | --- | --- | --- | --- | --- | --- | --- | --- | --- | --- | --- | --- |
| Characteristics | *circSTX6*-high | *circSTX6*-  low | n | *X²* | *P* |
| All cases | 34 | 46 |  |  |  |
| Gender |  |  |  | 1.047 | 0.3063 |
| Male | 24 | 37 | 61 |  |  |
| Female | 10 | 9 | 19 |  |  |
| Age(years) |  |  |  | 0.15 | 0.6986 |
| >60 | 9 | 14 | 23 |  |  |
| ≤60 | 25 | 32 | 57 |  |  |
| Tumor differentiation |  |  |  | 7.535 | 0.0061** |
| G1+G2 | 14 | 33 | 47 |  |  |
| G3 | 20 | 13 | 33 |  |  |
| Stage |  |  |  | 4.539 | 0.0331* |
| Ⅰ+Ⅱ | 25 | 42 | 67 |  |  |
| Ⅲ+Ⅳ | 9 | 4 | 13 |  |  |
| Tumor size |  |  |  | 10.55 | 0.0012** |
| >5 | 22 | 13 | 35 |  |  |
| ≤5 | 12 | 33 | 45 |  |  |
| AFP |  |  |  | 2.03 | 0.1542 |
| >400 μg/L | 18 | 17 | 35 |  |  |
| ≤400 μg/L | 16 | 29 | 45 |  |  |
| HBsAg |  |  |  | 0.3906 | 0.532 |
| Positive | 27 | 39 | 66 |  |  |
| Negative | 7 | 7 | 14 |  |  |

| **Table S4.** Target sequences of siRNAs and shRNAs used in this study | |
| --- | --- |
| Gene symbol | Targeted sequence |
| siCtrl | UUCUCCGAACGUGUCACGU |
| si*circSTX6*#1 | UCUCAUAUGACCAGUGGACAUGAAA |
| si*circSTX6*#2 | UAUGACCAGUGGACAUGAAAGAUCA |
| sh*circSTX6*#1 | TCTCATATGACCAGTGGACATGAAA |
| sh*circSTX6*#2 | TATGACCAGTGGACATGAAAGATCA |
| siHNRNPD#1 | AAUGUUGGUCUUAGUAAAUGU |
| siHNRNPD#2 | GUUGUAGACUGCACUCUGA |
| siATF3#1 | UAUCUGUUGGAUAAAGAGGUU |
| siATF3#2 | UCACAAAAGCCGAGGUAGC |
| siMETTL3#1 | GCUGCACUUCAGACGAAUU |
| siMETTL3#2 | GCAAGAAUUCUGUGACUAU |
| siMETTL14#1 | GCAGCACCUCGAUCAUUUA |
| siMETTL14#2 | GGAUGAAGGAGAGACAGAU |
| siWTAP#1 | CAGAUCUUAACUCUAAUG |
| siWTAP#2 | GCUUUGGAGGGCAAGUACAUU |
| siALKBH5#1 | ACAAGUACUUCUUCGGCGA |
| siALKBH5#2 | GCGCCGUCAUCAACGACUA |
| siFTO#1 | GGCAGAGATCCTGATAC |
| siFTO#2 | AUAGCCGCUGCUUGUGAGAUU |

| **Table S5.** Primer sequences used in in this study. | | |
| --- | --- | --- |
| Gene symbol | Forward primers | Reverse primers |
| *circSTX6*-divergent | CTGAAGAACATGTCCCAGCG | GATCTTTCATGTCCACTGGTCA |
| *circSTX6*-convergent | CACTGGAACAACAGATAAATATGGG | TGCTGCTCCTCAATGAAATGA |
| *circSTX6*-MeRIP-primer1 | GCTGGAGGAACAGGCAGTTA | TTGCAAGTTTCTTCATCACATTG |
| *circSTX6*-MeRIP-primer2 | GCTGGAGGAACAGGCAGTTA | CTTTTGCAAGTTTCTTCATCACA |
| *circSTX6*-MeRIP-primer3 | TCATCTGTGCAGGCATTAGC | GGTCCAGACGCCCATATTTA |
| *circSTX6*-MeRIP-primer4 | TGCAGGCATTAGCTGAAAGA | GGTCCAGACGCCCATATTTA |
| *circSTX6*-144aa | AAATATGGGCGTCTGGACCG | ATCTGATCTTTCATGTCCACTGGT |
| *STX6* | CTGCGGACTGTGAAGAATCA | CGTGCATTCTGAAATTGTGG |
| *GAPDH* | GGAGCGAGATCCCTCCAAAAT | GGCTGTTGTCATACTTCTCATGG |
| *GAPDH*-divergent | TGTACCATCAATAAAGTACCCTGTG | AAATCCGTTGACTCCGACCT |
| *U6* | GCTTCGGCAGCACATATACTAAAAT | CGCTTCACGAATTTGCGTGTCAT |
| *METTL3* | TTGTCTCCAACCTTCCGTAGT | CCAGATCAGAGAGGTGGTGTAG |
| *METTL14* | GAACACAGAGCTTAAATCCCCA | TGTCAGCTAAACCTACATCCCTG |
| *WTAP* | ACTGGCCTAAGAGAGTCTGAAG | GTTGCTAGTCGCATTACAAGGA |
| *ALKBH5* | CGGCGAAGGCTACACTTACG | CCACCAGCTTTTGGATCACCA |
| *FTO* | ACTTGGCTCCCTTATCTGACC | TGTGCAGTGTGAGAAAGGCTT |
| *HNRNPD* | GCGTGGGTTCTGCTTTATTACC | TTGCTGATATTGTTCCTTCGACA |
| *ATF3* | GTGCCGAAACAAGAAGAAGG | TCTGAGCCTTCAGTTCAGCA |
| *ANXA10* | TCAGAATGAACATGAAGTGGGG | TGGGCCACAACTTGCTGAC |
| *TKT* | TCCACACCATGCGCTACAAG | CAAGTCGGAGCTGATCTTCCT |
| *EPHA2* | TGGCTCACACACCCGTATG | GTCGCCAGACATCACGTTG |
| *AKR1B10* | TCAGAATGAACATGAAGTGGGG | TGGGCCACAACTTGCTGAC |
| *AQP3* | TTGGCTTTGCTGTCACTCTG | TATTCCAGCACCCAAGAAGG |
| *PHLDA1* | GAAGATGGCCCATTCAAAAGCG | GAGGAGGCTAACACGCAGG |
| *IL13RA2* | GGGCATTGAAGCGAAGATACA | GCCCAGGAACTTTGAACTTCTG |
| *ASPH* | CATGGAGGACACAAGAATGGG | CCAAACGACAGCTACAGATGT |
| *ETS2* | CCCCTGTGGCTAACAGTTACA | AGGTAGCTTTTAAGGCTTGACTC |
| *B3GNT5* | GGGCCTCGCTACCAATACTTG | CGGAACGTCGATCATAGTTTTCA |
| *ROBO1* | CGCCCCACACCCACTATTG | GAAGTCATCCCGAAGTATGGC |
| *NRG1* | CGGTGTCCATGCCTTCCAT | GTGTCACGAGAAGTAGAGGTCT |
| *HPD* | GAACCTCTAGCCTACAGGGG | TCTTTGTTCCAGGGGTTGAGC |
| *SULT1C2* | CAGCCTGCAACTGTGGACAA | GATGGCGGTGTTGGATGATG |
| *IL-6* | GCCCAGCTATGAACTCCTTCT | GAAGGCAGCAGGCAACAC |
| *IL-8* | GAGTGGACCACACTGCGCCA | TCCACAACCCTCTGCACCCAGT |
| *AFP* | CCAACAGGAGGCCATGCTT | GAATGCAGGAGGGACATATGTTT |
| *TNF* | CCTCTCTCTAATCAGCCCTCTG | GAGGACCTGGGAGTAGATGAG |
| *MYC* | GGCTCCTGGCAAAAGGTCA | CTGCGTAGTTGTGCTGATGT |
| *P16* | CCCCTGAGCTTCCCTAGTTC | ATATGCCTTCCCCCACTACC |
| *P21* | CCTGCCCAAGCTCTACCTT | AAGGCAGGAGATGTAGAGC |
| *CCNB1* | GAACCTGAGCCTGTTAAAGAAGA | CAGGTCTTCTTCTGCAGGGG |
| *CCND1* | GATCAAGTGTGACCCGGACT | TCCTCCTCTTCCTCCTCCTC |


**Table S6.** Antibodies used in this study.

| Antibody (Experimental use) | Dilution factor | Source | Identifier |
| --- | --- | --- | --- |
| HNRNPD (WB) | 1:1000 | ABclonal | Cat#A15679 |
| HNRNPD (RIP) | 5μg | ABclonal | Cat#A15679 |
| HNRNPD (IF) | 1:200 | ABclonal | Cat#A15679 |
| GAPDH (WB) | 1:1000 | Servicebio | Cat#GB15004 |
| m^6^A (MeRIP) | 2μg | EpigenTek | Cat#P-9018-24 |
| IgG (MeRIP) | 2μg | EpigenTek | Cat#P-9018-24 |
| ATF3 (WB) | 1:1000 | ABclonal | Cat#A13469 |
| ATF3 (IHC) | 1:200 | ABclonal | Cat#A13469 |
| METTL14 (IHC) | 1:200 | ABclonal | Cat#A8530 |
| METTL14 (RIP) | 5μg | Proteintech | Cat#26158-1-AP |
| Flag-tag (WB) | 1:1000 | Sigma-Aldrich | Cat#F3165 |
| Flag-tag (RIP) | 5μg | Sigma-Aldrich | Cat#F3165 |
| E-cadherin (IHC) | 1:500 | Servicebio | Cat#GB11082 |
| E-cadherin (WB) | 1:1000 | ABclonal | Cat#A20798 |
| N-cadherin (IHC) | 1:500 | Servicebio | Cat#GB11135 |
| N-cadherin (WB) | 1:1000 | ABclonal | Cat#A0433 |
| Vimentin (WB) | 1:2000 | ABclonal | Cat#A19607 |
| Snail (WB) | 1:1000 | ABclonal | Cat#A11794 |
| PCNA (IHC) | 1:500 | Servicebio | Cat#GB11010 |
| Ki67 (IHC) | 1:500 | Servicebio | Cat#GB111499 |
| STX6 (WB) | 1:1000 | Proteintech | Cat#10841-1-AP |
| circSTX6-144aa (WB) | 1:1000 | AtaGenix | Cat#21000800A-4-101421-A01 |
| circSTX6-144aa (IHC) | 1:500 | AtaGenix | Cat#21000800A-4-101421-A01 |
